# Acute pancreatitis: mechanisms and therapeutic approaches

**DOI:** 10.1038/s41392-025-02394-6

**Published:** 2026-01-14

**Authors:** Qian Hu, Yue Hu, Chunlu Tan, Yue Yang, Hang Su, Zixing Huang, Wenfu Tang, Rui Wang, Jingping Liu, Meihua Wan

**Affiliations:** 1 West China Center of Excellence for Pancreatitis, Institute of Integrated Traditional Chinese and Western Medicine, West China Hospital, Sichuan University, Chengdu, China; 2 Division of Pancreatic Surgery, Department of General Surgery, West China Hospital, Sichuan University, Chengdu, China; 3 Health Management Center, General Practice Medical Center, West China Hospital, Sichuan University, Chengdu, China; 4 Department of Radiology, West China Hospital, Sichuan University, Chengdu, China; 5 Department of Gastroenterology, West China Hospital, Sichuan University, Chengdu, China; 6 Department of Integrated Traditional Chinese and Western Medicine and NHC Key Laboratory of Transplant Engineering and Immunology, Frontiers Science Center for Disease-related Molecular Network, West China Hospital, Sichuan University, Chengdu, China; 7 First People’s Hospital of Shuangliu District, Chengdu, China

**Keywords:** Inflammation, Gastroenterology

## Abstract

Acute pancreatitis is a complex inflammatory condition characterized by sudden onset and rapid progression, with severe cases often associated with high mortality. In recent years, the global incidence of acute pancreatitis has been increasing, with marked regional differences. This increasing trend not only places a considerable burden on healthcare systems but also significantly affects the physical and psychological well-being of patients. The most common causes—gallstone disease, hypertriglyceridemia, and alcohol abuse—also vary by region. This review provides a structured summary of current knowledge regarding the definition and classification of acute pancreatitis, along with recent advances in clinical scoring systems, biomarkers, and predictive models based on artificial intelligence. These tools are particularly valuable for risk stratification and early clinical decision-making. In addition, this review discusses the multilevel pathophysiological mechanisms involved in acute pancreatitis, including aberrant enzymatic activation, calcium overload, impaired autophagy, inflammatory responses, and various forms of pancreatic acinar cell death. From a therapeutic perspective, both early-phase management and strategies for later disease stages are addressed. This review also briefly assesses adjunctive therapies rooted in traditional Chinese medicine, including bioactive monomers, compound herbal formulas, and external treatment modalities. Furthermore, attention is given to individualized treatment approaches for special populations, as well as to emerging therapeutic avenues such as nanotechnology and extracellular vesicle-based interventions. Together, these insights serve as a comprehensive reference for the diagnosis and management of acute pancreatitis while also suggesting potential directions for future research and innovation.

## Introduction

**Fig. 1:**
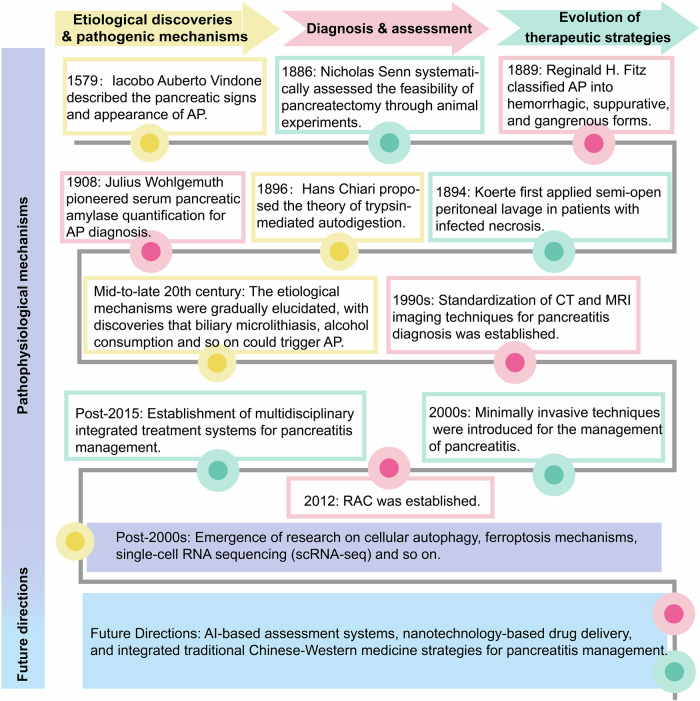
Historical evolution and future directions of acute pancreatitis treatment. This figure provides a systematic overview of research progress in AP from the 16th century to the present, covering its etiology, pathogenesis, diagnostic approaches, and therapeutic strategies. AI artificial intelligence, AP acute pancreatitis, CT computed tomography, MRI magnetic resonance imaging, RAC revised Atlanta classification, scRNA-seq single-cell RNA sequencing

Acute pancreatitis (AP) is one of the most common causes of abdominal pain in the emergency room. It is an inflammatory condition triggered by the inappropriate activation of pancreatic enzymes due to various factors, resulting in pancreatic self-digestion, edema, bleeding, and even necrosis of pancreatic tissue.^1^ In 1579, Iacobo Auberto Vindone was the first to describe the pancreatic signs and appearance of AP.^2^ Nearly three-quarters of a century later, in 1652, the Danish anatomist Nicolaes Pietersz Tulp provided a clinical description of AP.^3^ For several centuries thereafter, progress in understanding the disease has remained slow. A significant turning point occurred at the end of the 19th century and the beginning of the 20th century, when advancements in anatomical pathology led to a clearer understanding of AP. In a seminal article published in 1889, Reginald H. Fitz classified AP into hemorrhagic, suppurative, and gangrenous forms and described diffuse fat necrosis. He further identified pancreatic abscesses, splenic vein thrombosis, and pancreatic pseudocysts as related complications of the disease.^3^ Nicholas Senn, a contemporary of Reginald H. Fitz, emphasized the importance of surgical intervention for pancreatic diseases, highlighting the precision treatment of retention cysts and external pancreatic cyst formation. His contributions laid the groundwork for future experimental studies and surgical management of pancreatic conditions. In 1896, Hans Chiari investigated the pathophysiological mechanisms of AP and proposed the theory of trypsin-mediated autodigestion. However, it was not until the turn of the 20th century that the roles of gallstones and alcohol in the pathogenesis of AP were fully recognized. In addition to these two primary causes, a wide range of metabolic, autoimmune, parasitic, hereditary, and anatomical factors have since been implicated in AP. Despite this growing body of knowledge, approximately 10% to 15% of cases remain idiopathic and are categorized as acute idiopathic pancreatitis. In 1908, Julius Wohlgemuth at the Institute of Pathology of the Royal University of Berlin introduced a biochemical method to quantify serum pancreatic amylase for AP diagnosis.^2^ Since Kivisaari’s introduction of enhanced computed tomography (CT) imaging in 1984 to assess necrotizing pancreatitis and delineate the extent of pancreatic necrosis, CT has become instrumental in diagnosing, classifying, and monitoring treatment response in AP.^3^ Additionally, the Atlanta Conference in 1992 established globally accepted criteria for the classification and severity grading of AP, which were subsequently revised in 2012 (Fig. 1).

Although substantial advances in research have significantly improved the management of AP, approximately 25% of patients still progress to severe acute pancreatitis (SAP), which is characterized by complex pathological changes such as systemic inflammatory response syndrome (SIRS) and multiple organ dysfunction syndrome (MODS), with a mortality rate as high as 30–40%.^4^ Currently, the clinical management of SAP primarily involves supportive and symptomatic treatment, including fluid resuscitation, pain control, antibiotic therapy, enteral or parenteral nutrition, and preventive interventions targeting the underlying cause of AP.^5,6^ With the advancement of molecular and genetic technologies, the field of pancreatic research continues to expand, highlighting the importance of revisiting previous findings and lessons. This review provides a brief overview of the pathophysiology, clinical manifestations, diagnostic approaches, molecular mechanisms, and current treatment strategies for AP, with the aim of enhancing understanding and informing further research and clinical efforts to prevent disease progression.

## Epidemiology and etiology

### Prevalence, incidence, and mortality rates in different regions

According to global epidemiological data (cases per 100,000 people per year), the overall incidence of AP is 33.74 (95% CI: 23.33–48.81). A number of longitudinal studies have indicated that the global incidence of AP has been rising steadily over the past five decades. Among these, a meta-analysis based on average annual percentage changes from 1961 to 2016 reported an annual growth rate of 3.07%.^7,8^ This increasing trend appears to be driven by two main factors. First, the refinement of diagnostic criteria and advances in imaging technologies have substantially improved disease detection—particularly through the widespread adoption of the revised Atlanta classification (RAC) in North America, Europe, and the Asia–Pacific region,^9,10^ as well as the growing use of advanced imaging tools such as magnetic resonance cholangiopancreatography (MRCP) and endoscopic ultrasound (EUS).^11,12^ Second, shifts in the population-level distribution of risk factors have also played an important role; the underlying mechanisms will be discussed in detail later.

Epidemiological data indicate that the incidence of AP shows marked regional heterogeneity.^7,13,14^ North America and the Western Pacific are considered high-incidence areas, with rates in the United States being significantly higher than those in the Western Pacific. In contrast, Europe has maintained a relatively low incidence. Notably, high-quality, population-based epidemiological data remain scarce for South America, Africa, Southeast Asia, and the Eastern Mediterranean. With respect to temporal trends, different regions display distinct patterns: North America and Europe have shown a sustained increase, Asia has remained relatively stable, and South America and Oceania have exhibited a rising trend.^8^ In this context, conducting a detailed analysis of AP incidence characteristics across countries and continents remains highly meaningful. Researchers such as Roberts conducted a systematic analysis of hospitalized patients across 17 European countries between 1989 and 2015, revealing marked regional differences in the incidence of AP. The reported incidence rates ranged from 4.6 to 100 cases per 100,000 individuals. Among the surveyed regions, Eastern, Northern, and Central Europe—including Poland, Iceland, Germany, Norway, Spain, and northeastern England—exhibit the highest incidence rates.^14^ Notably, the majority of the epidemiological investigations included in this study (with the exception of two regional studies in Germany and one early study from Denmark) consistently indicated a continuous upward trend in AP incidence across Europe, with a median annual increase of 2.3%.^14^ However, it is important to interpret some findings with caution. For example, the data from Germany in Roberts’ study were derived exclusively from Lüneburg County,^15,16^ which had a limited sample size and apparent regional selection bias, thus potentially failing to represent the true national incidence of AP. Moreover, a recent cohort study of the Danish national population (1988–2018) also supported this increasing trend and highlighted significant sex differences: the incidence of AP in men rose by 29% (from 28.8 to 37.0 per 100,000 person-years), whereas the increase among women was more pronounced, reaching 148%, ultimately resulting in comparable incidence rates between the sexes.^17^ A retrospective cohort study from Sweden (1990–2013) further confirmed this pattern, reporting an increase in AP incidence from 25.2 to 38.3 per 100,000 person-years over the study period.^18^ Taken together, current epidemiological evidence supports the conclusion that the incidence of AP in Europe is increasing—a trend that appears to be echoed in data from other continents as well. A large-scale population-based study using administrative data from the Australian Institute of Health and Welfare reported a notable increase in the incidence of AP among adults, increasing from 37.56 to 52.09 cases per 100,000 person-years.^19^ In North America, particularly in the United States, the incidence of AP shows an increasing trend in recent years.^20^ Similarly, epidemiological patterns in South America indicate a gradual increase, albeit at a slower rate. For example, a retrospective study conducted at a tertiary medical center in Brazil (2001–2019) reported a modest increase in AP cases, from 255 to 277, which researchers attributed in part to potential referral bias.^21^ In Asia, the epidemiological profile appears more heterogeneous. A study from Zhabei District in Shanghai, China (population 810,000), reported a substantial increase in AP incidence, from 30.5 per 100,000 individuals in 2009 to 39.2 in 2014, corresponding to an average annual growth rate of 5.1%. In contrast, a meta-analysis by Jordan P. Iannuzzi et al., encompassing 44 studies conducted between 1961 and 2016, revealed that the overall incidence of AP across Asia has remained relatively stable, with an annual change of only 0.28%.^8^ This difference may partly reflect the impact of improved diagnostic capabilities and healthcare access in economically developed regions on disease detection rates.

In terms of population distribution, epidemiological data suggest that the incidence of AP does not differ significantly between males and females. However, certain subpopulations exhibit distinct patterns. For example, the incidence of AP in children is increasing rapidly—by approximately 5.44% per year—and is gradually approaching that reported in adults.^8,22^ In contrast, AP during pregnancy remains relatively rare, with an estimated incidence ranging from 1 in 1000 to 1 in 12,000 pregnancies.^23^ These differences may reflect variable exposure to risk factors across populations, as well as disparities in diagnostic capabilities. These nuances warrant careful consideration in both clinical practice and public health policymaking.

### Public health significance: economic and social impact

AP, a leading cause of hospitalization for gastrointestinal diseases, continues to pose a substantial clinical and economic burden on healthcare systems worldwide. For example, in the United States alone, there were 255,130 hospitalizations due to AP in 2021.^24^ Although advancements in diagnostic and therapeutic strategies have been made in recent years, the mortality rate associated with AP remains notable—1.0 per 100,000 people—resulting in 3441 deaths in 2021.^24^ This underscores the persistent strain that AP places on healthcare resources. In addition to its direct economic burden, AP also significantly affects patients’ social functioning and quality of life. Prolonged hospital stays and repeated interventions not only cause physiological harm but also contribute to considerable psychological stress.^25,26^ Emerging evidence suggests that even patients with mild AP (MAP) have at least twice the long-term risk of developing diabetes compared with the general population.^27^ Furthermore, among patients who experience a first episode of AP, 10%—and up to 36% of those with recurrent episodes—may develop chronic pancreatitis (CP).^28^ These findings emphasize that the burden of AP should not be evaluated solely in terms of its acute manifestations but must also account for its long-term health consequences. Therefore, future clinical and public health efforts should prioritize establishing a comprehensive AP registry encompassing the full disease course and population coverage. This system would allow for refined classification, long-term follow-up, and early identification of high-risk populations—particularly in areas of high incidence—thereby enabling more targeted prevention, control, and resource allocation strategies.

### Causes of acute pancreatitis

**Fig. 2:**
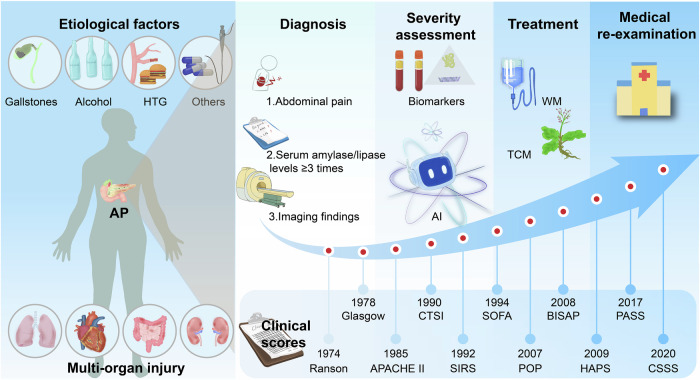
Overview of acute pancreatitis. This figure summarizes the current understanding of epidemiology, diagnosis, severity assessment, and treatment approaches. AI artificial intelligence, AP acute pancreatitis, APACHE II acute physiology and chronic health evaluation II, BISAP bedside index of severity in acute pancreatitis, CSSS Chinese simple scoring score, CTSI computed tomography severity index, HAPS harmless acute pancreatitis score, HTG hypertriglyceridemia, PASS pancreatitis activity scoring system, POP pancreatitis outcome prediction score, SIRS systemic inflammatory response syndrome, SOFA sequential organ failure assessment, TCM traditional Chinese medicine, WM western medicine

The etiological characteristics of AP exhibit substantial diversity and vary by region. Epidemiological evidence indicates that gallstones, hypertriglyceridemia (HTG), and alcohol abuse are the three leading causes of AP, with gallstone-induced pancreatitis being the most prevalent worldwide (Fig. 2).^29,30^ Gallstones can induce AP through two primary mechanisms. First, they may become lodged in the common bile duct or obstruct the pancreatic duct, resulting in elevated intraductal pressure and impaired pancreatic juice outflow.^31^ Second, gallstones can trigger inflammation of the bile duct, which subsequently affects the pancreas.^32^ Chronic excessive alcohol intake is recognized as the second major cause.^33^ Alcoholic pancreatitis involves complex dysfunction of both acinar and ductal cells within the pancreatic exocrine system. Alcohol exerts direct cytotoxic effects on the pancreas, and the development of AP in this context is multifactorial. For example, alcohol metabolites can promote enzyme synthesis and activate proteases, leading to local autodigestion of pancreatic tissue.^34^ Additionally, these metabolites interfere with pancreatic juice secretion by increasing protein precipitation and facilitating protein plug formation within the pancreatic ducts.^5^ Moreover, alcohol metabolites contribute to lipid droplet accumulation in the pancreas, activate the AMPK signaling pathway involved in lipid metabolism, and enhance autophagy in acinar cells.^35^ Alcohol can also inhibit apoptosis in acinar cells by upregulating antiapoptotic proteins, thereby promoting necrosis. It further impairs mitochondrial function by inducing hypophosphatemia, which contributes to organelle dysfunction.^36^ Although some studies suggest that moderate alcohol consumption may reduce the risk of AP, potential confounding effects from alcohol use in control groups cannot be ruled out,^37^ highlighting the need for more rigorously controlled studies to clarify this relationship. Recent research has also identified smoking as an independent risk factor for both acute and recurrent pancreatitis, with the risk being further increased when smoking is combined with alcohol consumption.^38,39^ HTG is recognized as a significant trigger of AP and represents its third leading cause.^40^ Approximately 15–20% of patients with severe HTG (serum triglyceride levels >1000 mg/dL) develop AP, often presenting with more severe clinical manifestations and a greater risk of persistent multiple organ failure.^41^ Currently, there are no established guidelines that clearly define the plasma triglyceride threshold associated with AP onset; however, the risk of AP increases in parallel with increasing serum triglyceride concentrations. When triglyceride levels exceed 11.3 mM, the risk of AP is approximately 5%, and when levels surpass 22.6 mM, the risk increases to 10–20%.^42^ Triglycerides themselves are not directly toxic to the pancreas, but free fatty acids generated through lipase-mediated hydrolysis can induce pancreatic inflammation, upregulate proinflammatory mediators, impair mitochondrial complex formation in acinar cells, and cause pathological increases in intracellular Ca²⁺ concentrations.^43^ Moreover, free fatty acids can aggregate into micelle-like structures with detergent properties, contributing to ischemia and local acidosis and ultimately triggering AP.^44^ Chang et al.^45^ reported that in a cohort of 46 patients with HTG-induced AP, 26.1% carried mutations in the cystic fibrosis transmembrane conductance regulator gene. Subsequent studies using two HTG mouse models demonstrated that, compared with control mice, HTG mice presented delayed pancreatic regeneration, marked by impaired morphological restoration and disrupted cellular responses, including fibrotic inflammation and acinar cell proliferation.^45^ Clinical follow-up data further indicated that, in patients with SAP, elevated serum triglyceride levels were associated with decreased fecal elastase-1 levels.^46^ In addition to the three major causes, various medications have been implicated in the development of AP, although drug-related cases account for less than 5% of all cases.^47,48^ Iatrogenic injury is another recognized contributor, most commonly following manipulation of the ampulla or pancreatic duct during procedures such as endoscopic retrograde cholangiopancreatography (ERCP), which carries an estimated risk of 10%,^49^ or more rarely after EUS, with a risk of less than 1%.^50^ Moreover, comorbid conditions such as obesity and type 2 diabetes are frequently associated with an increased risk of pancreatitis.^33,51^ Additionally, a proportion of cases with no obvious cause are classified as idiopathic pancreatitis.^52^

The etiological characteristics of AP were then analyzed. The increase in biliary AP is significantly correlated with the increasing global prevalence of biliary diseases, obesity, and diabetes.^53,54^ The distribution of alcoholic AP reflects regional drinking patterns, remaining stable or declining in North America while continuing to rise in Southeast Asia and China.^55–57^ AP induced by HTG has distinct seasonal variations and is strongly associated with increased consumption of high-fat diets during holiday periods.^58,59^ Region-specific analyses further highlight substantial variation in the etiological spectrum across different geographic areas. In parts of Eastern and Northern Europe, the incidence of biliary AP is comparable to or slightly exceeds that of alcoholic AP.^60–62^ In southern Romania, most patients are male, and alcohol is the predominant etiological factor.^63^ In Mediterranean and Southern European countries, gallstones are the leading cause. In contrast, Northern and Western European countries tend to exhibit more balanced etiological distributions.^14,64,65^ Drug-induced AP and other risk factors, such as smoking, are receiving increasing attention. Notably, systematic reviews in Western populations have revealed a significant association between increased cannabis use and AP incidence.^66,67^ In North America, gallstones and alcohol remain the leading causes.^68^ In South America, data from Chile show marked sex differences: biliary AP is more common among females, whereas alcoholic AP accounts for 17% of male cases and is rare in females.^69^ In Oceania, alcoholic pancreatitis is most frequently observed in younger Australian-born males, whereas biliary AP tends to affect older females.^70,71^ In Asia, alcoholic AP predominates in India and is largely restricted to males, mirroring regional drinking patterns.^72^ Southern Israel presents a distinct etiological profile: gallstone-related AP is most common, and drug-induced AP is markedly more common than in other regions and is associated primarily with hydrochlorothiazide and GLP-1 receptor agonists.^73^ In East Asia, substantial variation exists: in China, biliary AP is predominant and may progress to severe sepsis,^58,59^ whereas alcohol plays a secondary role^74,75^; in contrast, alcohol is the leading cause of AP in Japan.^76^

In terms of population distribution, the incidence rates of biliary and alcoholic AP are generally similar between males and females.^18,53,77,78^ However, their age distributions differ significantly: biliary AP is more common in elderly individuals, whereas alcoholic AP is more common in individuals aged 35–44 years.^14^ Notably, the clinical recognition of mixed-etiology AP—particularly involving biliary factors, HTG, and alcohol—has steadily increased, especially among younger individuals with concurrent fatty liver and metabolic abnormalities.^79^ Nevertheless, the underlying pathological mechanisms remain poorly understood. The etiological spectrum of AP also varies across special populations. In pediatric patients, the predominant causes include biliary/obstructive factors, drug-induced AP, and systemic diseases.^80–82^ Among these, biliary obstruction in children is frequently caused by biliary stasis, which differs fundamentally from common causes in adults, such as gallstones or tumors.^80,83^ Similarly, the etiology of AP in children shows notable regional variation. Gallstone disease is the most common etiology in Asia, although idiopathic pancreatitis is the predominant etiology among children in Dubai and Lebanon,^84,85^ trauma in Oceania, and idiopathic AP in Europe and North America.^86,87^ AP during pregnancy is relatively rare, with biliary disease and hyperlipidemia as the leading causes. The latter is closely associated with the physiological hyperlipidemia characteristic of pregnancy, during which serum triglyceride levels can increase two- to threefold compared to pre-pregnancy levels.^23^

The above content provides a systematic overview of the major etiological factors, pathogenic mechanisms, and epidemiological characteristics of AP across various regions and populations, offering valuable clinical insights. First, with the increasing prevalence of metabolic syndrome, nonalcoholic fatty liver disease, and other related conditions—along with the accumulation of multiple risk factors in individuals—the clinical detection of mixed-type AP is increasing. However, systematic studies on its underlying mechanisms, prognostic features, and intervention strategies remain limited. Future research should prioritize etiological diagnosis and mechanistic exploration in this subgroup, with particular attention given to the pathophysiological changes associated with metabolic disorders. Second, the etiological heterogeneity observed in pediatric and pregnant populations underscores the need for more individualized prevention and management approaches. For example, the proportion of AP caused by drug reactions and systemic diseases is substantially greater in children than in adults, whereas AP during pregnancy is more often associated with hyperlipidemia and biliary pathology. The etiology, diagnostic pathways, treatment options, and prognostic indicators in these groups differ significantly from those in the general population. Therefore, the development of population-specific diagnostic criteria, treatment standards, and long-term follow-up protocols is urgently needed. Third, regional variation in the etiological spectrum of AP highlights the importance of building localized disease surveillance and intervention frameworks. For example, biliary AP is predominant in Mediterranean countries, whereas alcoholic AP appears to be on the rise in parts of South Asia, pointing to a need for public health strategies that consider local dietary patterns, lifestyle habits, and genetic predispositions. Additionally, while several studies have identified key pathogenic mechanisms underlying biliary, alcoholic, and HTG-related AP, most investigations remain focused on isolated signaling pathways or specific cellular subsets. There is a critical need for integrative research grounded in systems biology. A combined application of transcriptomic, metabolomic, and immunomic approaches is recommended to investigate the similarities and differences in local and systemic inflammatory networks in the pancreas across distinct etiological contexts. These findings may help establish a molecular foundation for designing more targeted therapeutic strategies.

## Clinical features and prognostic evaluation

### Definition, clinical manifestations, and diagnosis

AP is an inflammatory condition resulting from the premature activation of pancreatic enzymes within the pancreas, triggered by various etiologies.^1^ Clinically, the most prominent symptom is sudden-onset, persistent, and severe upper abdominal pain, which is reported as the initial complaint in approximately 80–95% of patients.^88,89^ This pain often radiates to the back and tends to worsen with food intake, fluid consumption, or lying supine. It is frequently accompanied by nausea, vomiting, and mild to moderate fever.^89,90^ On physical examination, abdominal distension and reduced bowel sounds are common, whereas rebound tenderness, an important feature in distinguishing AP from other causes of acute abdomen, is relatively rare.^90^ A thorough medical history should assess prior episodes of AP, gallstone disease, alcohol use, family history of pancreatitis, and recent infections, trauma, or medication exposure, as these factors are key to identifying the underlying etiology.^90^

The internationally accepted diagnostic criteria for AP are based on the 2012 RAC, which requires the presence of at least two of the following three features: (1) characteristic abdominal pain; (2) serum lipase or amylase levels ≥ three times the upper limit of normal; and (3) characteristic findings on imaging (CT or magnetic resonance imaging [MRI]) (Fig. 2).^9,91,92^ This diagnostic standard has been widely endorsed by both domestic and international clinical guidelines. In laboratory testing, current diagnostic strategies increasingly favor serum lipase over traditional amylase, owing to its higher sensitivity and specificity.^93,94^ This enzymatic elevation reflects the underlying pathology: pancreatic acinar cell injury leads to the release of enzymes into the interstitial space, which are subsequently absorbed into the bloodstream.^93–95^ Notably, the lipase/amylase ratio can assist in distinguishing alcoholic pancreatitis, while elevated transaminase levels are more suggestive of a biliary origin.^31^ Detection of urinary trypsinogen-2 also offers a distinct advantage in ruling out the disease, given its high negative predictive value of up to 99%.^93,96,97^ In addition, although emerging markers such as serum immunoreactive trypsin and chymotrypsin show theoretical potential, their clinical utility remains to be validated through further studies.^98^ Imaging modalities play a pivotal role in the diagnosis and staging of AP. Abdominal ultrasound is valuable in the early evaluation phase, as it helps identify potential causes, such as biliary stones, and monitor peripancreatic fluid collections (PFCs).^99^ However, its diagnostic utility can be significantly limited by factors such as intestinal gas or obesity.^99^ Contrast-enhanced CT remains the preferred imaging modality for AP,^92^ offering excellent diagnostic sensitivity and specificity^100^ and providing clear visualization of the pancreatic parenchyma and surrounding structures.^101^ New techniques—including dual-energy CT, spectral CT, subtraction CT, and perfusion CT—have shown promise in detecting necrosis earlier than conventional CT, but their clinical application is still limited. Although MRI is used less frequently than CT is, it offers distinct advantages: it is suitable for patients with iodine contrast allergies, for characterizing peripancreatic fluid, and for assessing the pancreatic duct, particularly in suspected cases of ductal rupture.^102^ However, early imaging may underestimate the extent of necrosis, as lesions may not yet be fully developed; therefore, routine early CT is not recommended for patients with a confirmed and stable diagnosis.^9^ MRI and ultrasound may serve as adjunctive tools in selected cases but are generally not considered first-line imaging modalities.^103,104^

In the diagnostic practice of AP, several key considerations warrant particular attention. At the clinical research level, accurately documenting the time of disease onset is essential and should be based on the onset of abdominal pain rather than the time of hospital admission, with a detailed record of the interval from symptom onset to the initial medical evaluation.^9^ From a laboratory diagnostic perspective, although both amylase and lipase are important biomarkers, lipase has a longer half-life, offering an advantage in diagnosing patients who present late.^92^ However, clinicians should be aware that various gastrointestinal (e.g., perforated peptic ulcer, intestinal obstruction) and non-gastrointestinal conditions (e.g., renal insufficiency, macroamylasemia) can also cause mild elevations in pancreatic enzyme levels.^105^ Therefore, a thorough differential diagnosis is necessary in patients with mildly elevated enzymes or atypical clinical presentations. When there is a high degree of clinical suspicion of AP but serum enzyme levels do not meet diagnostic thresholds, especially in patients with delayed presentation, imaging modalities such as contrast-enhanced CT or MRI are crucial for diagnosis.^104,106^ Furthermore, EUS has demonstrated superior sensitivity compared conventional imaging modalities (e.g., CT, MRCP) in detecting biliary pancreatitis, particularly in idiopathic cases. EUS can identify underlying etiologies such as microlithiasis or pancreatic duct strictures in 60–70% of cases. A meta-analysis comparing EUS and MRCP in the diagnosis of idiopathic AP reported diagnostic rates of 60% for EUS, 24% for MRCP, and 43% for secretin-enhanced MRCP. These findings suggest that EUS should be the preferred diagnostic modality for patients with acute or recurrent pancreatitis of unknown origin.^107,108^

### Classification of acute pancreatitis

The clinical course of AP is typically divided into two phases: an early phase (≤2 weeks from onset) and a late phase ( > 2 weeks), each associated with a distinct peak in mortality. The disease can evolve from a self-limiting mild form to a severe condition characterized by MODS. To standardize clinical practice, the RAC and determinant-based classification systems have been widely adopted internationally as standard frameworks for assessing disease severity.^9,109^ The RAC, an improvement on the Marshall scoring system, quantitatively evaluates the function of the respiratory, cardiovascular, and renal systems. On the basis of the duration of organ failure (defined as lasting >48 h) and the presence of local complications (e.g., pancreatic necrosis or peripancreatic effusion), AP is categorized into three grades: mild (no organ failure or local complications), moderately severe (transient organ failure or local complications) (MSAP), and severe (persistent organ failure).^9,109^ This classification not only reflects the pathophysiological basis of AP but also provides a practical tool for guiding clinical intervention. Further investigations have identified two broad categories of organ failure in AP: early primary organ failure, which occurs within 72 h due to systemic inflammation, and late secondary organ failure, which typically develops after one week and is often linked to infectious pancreatic necrosis. The therapeutic window for early failure is narrow (24–48 h), whereas intervention for late failure may last several days to weeks.^110^ The complications of AP are categorized into systemic complications and local complications. Systemic complications refer to new-onset organ failure (involving the respiratory, cardiovascular, or renal systems) or acute exacerbation of pre-existing comorbidities. Local complications include peritoneal fluid accumulation, pseudocysts, and pancreatic or peripancreatic necrosis. Among all complications, persistent organ failure (lasting more than 48 h) is the most important predictor of adverse outcomes. While the overall mortality rate for AP is approximately 2%, it increases substantially to approximately 30% when persistent organ failure is present.^9,109^ Notably, early deaths (within the first week) are most often attributed to ongoing and worsening organ dysfunction. The co-occurrence of persistent organ failure and infected pancreatic necrosis (IPN) further elevates the risk of mortality.^109^

In terms of pathological morphology, AP is broadly categorized into two types: necrotizing (typically corresponding to moderate to severe cases) and interstitial edematous (mostly mild cases).^9,111^ The interstitial edematous type is the most common type and is characterized by diffuse—or occasionally localized—enlargement of the pancreas with inflammatory edema. Contrast-enhanced CT typically reveals relatively uniform enhancement of the pancreatic parenchyma, with surrounding peripancreatic fat showing hazy or mildly linear inflammatory changes and, in some cases, associated peritoneal effusion. The clinical symptoms of these patients generally resolve within one week, and the prognosis is favorable.^112^ In contrast, necrotizing pancreatitis—although observed in only 5–10% of cases—carries significant clinical consequences.^113–115^ It is defined by the presence of diffuse or focal non-perfused areas exceeding 3 cm or involving more than 30% of the pancreatic volume.^113–115^ Further analysis revealed that approximately 45% of necrotizing pancreatitis cases involve only peripancreatic necrosis, another 45% involve both pancreatic and peripancreatic necrosis, while isolated pancreatic parenchymal necrosis accounts for only approximately 5% of cases.^113–115^ Importantly, even in cases of isolated peripancreatic necrosis, where the pancreatic parenchyma appears normal on imaging, the rate of complications and need for intervention remain significantly higher than those in interstitial edematous pancreatitis.

These classification systems provide valuable guidance for clinical decision-making by clearly delineating the duration of organ failure (persistent or transient) and the characteristics of local complications. However, these methods are primarily applicable to assessing disease severity in later stages and offer limited utility for early prediction or timely intervention. Consequently, the ability to evaluate disease severity and anticipate outcomes at an earlier stage is critical. Early identification may support prompt treatment, help prevent progression to severe forms, reduce mortality, and ultimately improve patient prognosis.

### Severity assessment in acute pancreatitis

#### Clinical scoring system

Currently, tools for the early prediction of AP severity include clinical scoring systems such as acute physiology and chronic health evaluation II (APACHE II), SIRS, and the modified Glasgow score, as well as imaging-based assessments such as the CT Severity Index (CTSI) and Balthazar score (Fig. 2 and Table 1).^116,117^ Among traditional scoring systems, the Ranson criteria (1974) and Glasgow-Imrie score (1978) are considered first-generation predictive tools. Although these systems are disease-specific, they require the collection of multiple clinical and laboratory parameters within 48 h of admission, making them cumbersome and complex to apply.^118–120^ For example, the Ranson criteria involve 11 indicators—five assessed at admission and six assessed within 48 h—necessitating repeated evaluations and calculations by physicians.^118,120^ The APACHE II score, introduced in 1985 as a general critical illness assessment tool, is widely used to evaluate AP severity. However, its reliance on 12 physiological variables—including temperature, mean arterial pressure, heart rate, respiratory rate, oxygenation index, arterial pH, serum sodium, potassium, creatinine, hematocrit, white blood cell count, and Glasgow Coma Scale score—can make it impractical for non-intensive care unit (ICU) settings.^120,121^ In the early 1990s, the CTSI, which is based on the Balthazar grading system (grades A to E) and the extent of pancreatic necrosis, was introduced to improve the radiological assessment of AP severity. Later, the Modified CTSI was developed by incorporating extrapancreatic complications to further enhance the accuracy of severity evaluation.^122^ The SIRS criteria (1992) have gained attention for their simplicity and high sensitivity; meeting any two of the criteria is sufficient for diagnosis. Studies have shown that the presence of SIRS within 24 h of AP onset predicts organ failure with 85% sensitivity and mortality risk with up to 100% sensitivity.^123,124^ If SIRS persists beyond 48 h, its specificity for poor prognosis significantly increases, and such patients should be considered for ICU transfer. Moreover, early reversal of SIRS and timely intervention for organ dysfunction are crucial for improving outcomes.^10,125,126^ Despite its sensitivity in predicting early organ failure and mortality, SIRS lacks specificity for SAP, making it more suitable as a screening tool than as a diagnostic tool.^126–128^ The Sequential Organ Failure Assessment score, developed in 1994, includes six variables and was originally designed for assessing MODS in SAP. However, its disease specificity for AP is limited.^129^ The Pancreatitis Outcome Prediction score, introduced in 2007, also includes six parameters. It is simple and quick to use in clinical practice and is especially helpful for stratifying biliary AP, although clinical validation remains limited.^130^ The Bedside Index of Severity in Acute Pancreatitis (BISAP), developed in 2008, represents an effort to simplify traditional scoring systems. It relies on five readily assessable variables (blood urea nitrogen (BUN) > 25 mg/dL, altered mental status, SIRS, age >60 years, and pleural effusion) and has shown strong predictive value for in-hospital mortality, with a BISAP score of 5 associated with a mortality rate exceeding 20%.^9,120,131,132^ A BISAP score ≥3 correlates strongly with adverse outcomes such as organ failure, persistent organ failure, and pancreatic necrosis. Its simplicity has made it widely applicable in both emergency and general ward settings.^131,133^ The Harmless Acute Pancreatitis Score (HAPS), introduced in 2009, consists of three parameters and is specifically designed to identify patients who are unlikely to require intensive care.^134^ Although both the BISAP and HAPS are user-friendly, their sensitivity and specificity remain suboptimal.^123,124^ The Pancreatitis Activity Scoring System (PASS), introduced in 2017, integrates both objective and subjective metrics to predict mortality and organ failure. However, clinical data on its utility are still limited.^135^ In 2020, the Chinese Simple Scoring System was developed and has demonstrated relatively high accuracy in predicting infection in AP patients, although studies evaluating its performance remain scarce.^135^

**Table 1:** Clinical scoring system for the early prediction of acute pancreatitis severity

Scoring system	Scoring content	Advantage	Disadvantage	Application	Ref.
1974-Ranson	Eleven indexes (five assessed at admission and six within 48 h), such as age, WBC, LDH, blood sugar, BUN, HCT, etc.	1. Have the longest history and is widely used in clinic; 2. Multiple clinical and laboratory parameters make it possible to consider patients more comprehensively, thus improving the reliability of prediction; 3. The parameters of 48 h after admission can reflect the dynamic changes of patients’ condition	1. Need to be evaluated twice (at admission and 48 h after admission), and the operation is complicated; 2. Do not evaluate organ failure	Early prediction of SAP and mortality	^118^
1978- Glasgow Imrie	Eight indicators (evaluated within 48 h), such as PaO₂, albumin, serum calcium, etc.	The high specificity for evaluating the incidence of serious diseases	Early prediction ability is limited	Predict severe AP	^120^
1985-APACHE II	12 physiological indexes, age and history of chronic diseases	1. The sensitivity of predicting the severity of AP is the highest; 2. Predicting the prognosis of SAP has the strongest predictive ability	Complex (multiple indicators need to be calculated)	Predict the severity of AP and the prognosis of SAP	^120^
1990- CTSI	Based on CT classification (A-E) and necrosis range	Evaluating AP patients shows superior performance in terms of mortality, persistent organ failure, and local complications, and can intuitively assess pancreatic necrosis and complications	Early ( < 48 h) assessment may underestimate necrosis	Imaging evaluation of necrosis, infection risk, and surgical indications	^122^
1992- SIRS	1. Body temperature > 38 °C or <36 °C; 2. Heart rate >90 beats/min; 3. Respiratory frequency > 20 beats/min or PaCO_2_ < 32 mmHg (1 mmHg=0.133 kPa); 4. The total number of WBC is more than 12×10^9^/L or less than 4×10^9^/L, or the proportion of immature neutrophils is more than 10%. Those with two or more of the above conditions are diagnosed as SIRS	High sensitivity in predicting early organ failure and mortality	Require dynamic monitoring	Lack specificity for SAP, making it more suitable as a screening rather than a diagnostic tool	^127,128^
1994- SOFA	Six organ function indexes (respiration, coagulation, liver, circulation, CNS, kidney)	In patients with septic shock, the predictive ability of the SOFA score is better than that of other scores	Require dynamic monitoring	Severity Assessment of SAP Complicated with MODS	^129^
2007-POP	Six indicators: age (years), mean arterial pressure (mmHg), PaO₂/FiO₂, pH value, urea (mg/dl) and calcium (mg/dl)	1. Cheap, simple, rapid and high specificity; 2. Stratified patients with biliary AP, and timely guided medical management according to the severity	Less research, not widely verified	As a prognostic indicator	^130^
2008- BISAP	Five simple indicators (SIRS, altered mental status, age, BUN and pleural effusion)	Simple and quick, high specificity in predicting adverse outcomes such as organ failure, persistent organ failure, and pancreatic necrosis	1. Lower sensitivity; 2. The collection of imaging data is prone to omissions	Early screening of SAP and mortality risk	^120,132^
2009- HAPS	Three indicators: the patient’s physical examination results, as well as routine laboratory measurements of hematocrit and serum creatinine	Minimalism (assessment upon admission)	Only MAP is identified, and SAP is not predicted	Rapid and preliminary identification of patients with AP without intensive care	^134^
2017- PASS	Includes objective items (organ failure and SIRS) and subjective items (abdominal pain, morphine uses and the ability to tolerate solid diet)	The best predictor of mortality and organ failure, especially ARDS	Clinical data on its utility are still limited	Predict mortality and organ failure	^135^
2020- CSSS	Six variables: including serum creatinine, blood sugar, lactate dehydrogenase, heart rate, CRP and degree of pancreatic necrosis	The prediction accuracy of pancreatic infection is the highest	No research to evaluate CSSS, and research with larger sample size and prospective design is needed to verify it	Predict SAP severity and infection	^135^

*AP* acute pancreatitis, *APACHE II* acute physiology and chronic health evaluation II, *ARDS* acute respiratory distress syndrome, *BISAP* bedside index of severity in acute pancreatitis, *BUN* blood urea nitrogen, *CNS* central nervous system, *CRP* C-reactive protein, *CSSS* Chinese simple scoring system, *CTSI* CT severity index, *HAPS* harmless acute pancreatitis score, *HCT* hematocrit, *LDH* lactate dehydrogenase, *MAP* mild acute pancreatitis, *MODS* multiple organ dysfunction syndrome, *PASS* pancreatitis activity scoring system, *POP* pancreatitis outcome prediction score, *SIRS* systemic inflammatory response syndrome, *SOFA* sequential organ failure assessment score, *WBC* white blood cell

By evaluating the predictive performance and clinical utility of various scoring systems, several notable features can be identified. First, in terms of predictive accuracy, the Glasgow Imrie score, developed specifically for AP, has relatively high sensitivity and specificity.^120^ Some researchers argue that although the Ranson score is one of the earliest scoring systems, it has retained clinical value over time, especially because of its emphasis on early assessment and dynamic monitoring.^136^ Some researchers have reported that the Ranson score demonstrates predictive performance comparable to that of the BISAP score, with higher sensitivity but lower specificity.^137^ These findings suggest that the Ranson score remains a valuable tool for evaluating disease severity and prognosis in patients with AP, particularly when time or resources are limited. The BISAP score, meanwhile, offers a favorable balance between simplicity and predictive capability.^116,117,133^ It is regarded by some researchers as a reliable tool for estimating mortality risk in AP patients, especially in primary care settings where medical resources are limited. In such contexts, it can facilitate the early identification of high-risk patients and support timely referrals to tertiary care centers.^138^ However, although the SIRS criteria exhibit high sensitivity, their specificity remains relatively low, at approximately 41%.^123,124^ In terms of clinical applicability, both the HAPS and BISAP are better suited for use in emergency departments and general wards because of their simplicity and accessibility of the required parameters.^123,124,133^ Gupta et al.^139^ noted that the HAPS is easy to calculate, feasible for routine use at admission, and based on readily available clinical data. Compared with other systems, it has particular value in identifying mild cases of AP, offering superior triage performance. This makes it a promising tool for early, cost-effective management and suggests its potential as a clinical reference standard. By contrast, the APACHE II score, owing to its complexity, is more applicable in ICU settings.^121^ Systematic reviews have indicated that among existing tools, the APACHE II score and BISAP score provide relatively strong predictive performance.^116,117^ Although several CT-based scoring systems (e.g., the Balthazar score and CTSI) provide objective, quantitative metrics, radiographic signs of severe lesions often lag behind clinical symptoms.^140^ For example, multiple studies have demonstrated that contrast-enhanced CT performed within 72 h of symptom onset may significantly underestimate the extent of pancreatic necrosis or misclassify disease severity,^9,141,142^ primarily due to the dynamic progression of inflammatory changes. Therefore, for patients with suspected moderate to severe AP, delaying definitive CT evaluation—ideally until 5 to 7 days after symptom onset—is generally recommended to achieve a more accurate radiological assessment. In addition, CT scoring systems and clinical scoring tools have shown similar performance in stratifying disease severity, yet both have demonstrated limited utility in guiding early treatment decisions and predicting final clinical outcomes.^113,115,143^ This inconsistency underscores a critical gap between the current predictive tools and their role in facilitating timely clinical intervention.

The above-mentioned scoring tools generally have limitations such as computational complexity and insufficient timeliness. Notably, early interventions guided by predictive scoring—such as nutritional support or prophylactic antibiotics—have not shown clear clinical benefit.^144,145^ In addition, while current scoring systems can identify mild cases with high accuracy, their ability to predict moderate to severe AP remains limited, with an accuracy of approximately 50%.^146,147^ Another critical limitation is the delay in predictive utility: the optimal scoring time point often occurs more than 48 h after symptom onset, at which time clinical signs of SAP are typically evident, potentially missing the window for effective early intervention.^133,148^ This has spurred interest in real-time assessment strategies^149^ and highlights the need for novel biomarkers capable of reliably predicting disease progression early during hospitalization. In summary, it is important to recognize several inherent limitations of current scoring systems. Crucially, these tools function more as “descriptors of disease severity” than as true predictors; they tend to reflect rather than anticipate the clinical trajectory.^91^ Moreover, no high-quality evidence currently demonstrates that predictive scoring systems improve patient outcomes in a meaningful way,^150^ highlighting a persistent gap between prediction and intervention.

#### Biomarkers

In the field of biomarkers, elevated serum creatinine and hematocrit are indicators of fluid loss and inadequate intravascular volume; these indicators are useful not only for assessing disease severity but also for monitoring the response of volume-depleted patients to intravenous fluid therapy.^92,125^ In recent years, BUN has shown promise in predicting the severity and mortality of SAP. An increase in BUN within the first 24 h is now recognized as a significant risk factor for mortality and is closely associated with multiple organ failure and severe disease progression.^151^ Additionally, novel hematological indexes, such as the neutrophil-to-lymphocyte ratio, platelet-to-lymphocyte ratio, and red blood cell distribution width (RDW), have demonstrated considerable predictive value. Jagodić Ejubović et al.^152^ proposed that the RDW-to-platelet ratio (RDW/PLT) may serve as a valuable biomarker for assessing disease severity. C-reactive protein (CRP), a routinely used inflammatory marker, has a positive predictive value of 31.7% for SAP when levels reach ≥190 mg/L within 48 h of admission.^153^ Other inflammatory markers, such as interleukin-6 (IL-6) and procalcitonin, exhibit more substantial elevations and prolonged persistence in patients with SAP. Importantly, while elevated serum amylase and lipase levels are key diagnostic criteria, they do not correlate with disease severity.^91^ Advances in molecular biology have led to the identification of novel biomarkers with promising predictive potential, including hepatocyte growth factor, angiopoietin-2, interleukin-8 (IL-8), soluble tumor necrosis factor receptor (TNFR) 1 A, and activin A.^154,155^ Furthermore, recent findings on the immune checkpoint molecule B7-H5 have revealed its clinical relevance in SAP. Specifically, low membrane expression of B7-H5 is significantly negatively correlated with several indicators of disease severity, such as elevated hematocrit, BUN, creatinine, and lactate levels and increased Ranson and APACHE II scores,^156^ highlighting the potential role of innate immunity-mediated downregulation of the membrane expression of B7-H5 in immune dysregulation during SAP. These findings suggest that B7-H5 could serve as both a predictive biomarker and a potential therapeutic target. In parallel, genetic polymorphism analyses are offering new approaches for early risk assessment.^157^ For example, a European multicenter study involving 424 patients with AP used gene set enrichment analysis to identify enhanced short-chain fatty acid production pathways in the oral microbiome of patients with severe disease, suggesting potential novel biomarkers and therapeutic targets.^158^ Experts increasingly emphasize that biomarkers should be interpreted alongside established scoring systems to enhance prognostic accuracy. For example, nucleated red blood cells—detected in approximately 10% to 30% of critically ill patients—have been closely associated with elevated in-hospital mortality.^159,160^ Building on this observation, Xu et al.^161^ reported that the qualitative detection of peripheral nucleated red blood cells, when combined with the Charlson Comorbidity Index and the APACHE II score, may serve as a useful tool for predicting the prognosis of SAP and for informing early, individualized treatment strategies in high-risk patients. Despite these advances, accurately predicting disease progression within the first 24 to 48 h of admission remains a major clinical challenge.

### Artificial intelligence-based predictive models and risk stratification tools

Conventional scoring systems for AP have inherent limitations in accurately predicting disease severity and clinical outcomes. These shortcomings are particularly pronounced in the early phase of the disease, where limited predictive accuracy, narrow temporal applicability, and inadequate discriminatory power constrain their clinical utility.^148^ Such limitations may delay the implementation of timely and appropriate management strategies. In recent years, artificial intelligence (AI)—especially machine learning (ML)—has emerged as a promising tool to address these challenges.^162^ By recognizing complex patterns within high-dimensional clinical datasets, ML models offer improved capabilities for risk stratification and outcome prediction in AP. This section provides a critical overview of representative high-impact studies published between 2019 and June 2025, summarizing model architectures, performance metrics, key predictive features, translational barriers to clinical adoption, and potential future directions in the field.^163,164^

#### Machine learning algorithms and comparative performance

A diverse range of ML algorithms has been explored and applied for risk prediction in AP. These methods span traditional statistical methods, such as logistic regression, support vector machines, and decision trees, to more sophisticated ensemble techniques, such as random forests and gradient boosting machines (e.g., XGBoost), as well as deep learning architectures, including artificial neural networks and convolutional neural networks (CNNs).^165^ Among these, ensemble models—particularly random forest (RF) and XGBoost—have consistently demonstrated strong performance when applied to structured, tabular clinical datasets. RF models are frequently favored because of their ability to model non-linear relationships and complex feature interactions, alongside their robustness and built-in feature importance metrics.^164^ Similarly, XGBoost is widely used for its computational efficiency and high predictive accuracy. For example, an XGBoost model developed by Thapa et al.^166^ using a large U.S. database achieved an area under the receiver operating characteristic curve (AUROC) of 0.921 for predicting SAP requiring ICU admission, significantly outperforming conventional scoring systems such as the BISAP and HAPS. Furthermore, an interpretable XGBoost model achieved strong performance in predicting ICU mortality among AP patients, with an external validation AUROC of 0.89, surpassing established metrics such as the APACHE IV score and the Sequential Organ Failure Assessment score.^167^ Moreover, DL techniques—particularly CNNs—offer distinct advantages for analyzing medical images. Studies combining abdominal CT scans with clinical data via CNN-based models have reported high accuracy in classifying AP severity. One such hybrid model, which integrates CNN-derived imaging features with clinical variables, achieved an AUROC of 0.920 in an external validation cohort for predicting SAP, with high sensitivity (90.3%) and specificity (82.9%), markedly outperforming models based solely on clinical or imaging data.^168^ In addition, deep learning models such as gated recurrent units have shown promise in capturing the temporal progression of AP. Luo et al., ^169^ for example, applied a multi-task gated recurrent unit to a large Chinese cohort (n = 13,645) for real-time prediction of organ failure, including respiratory failure, circulatory failure, and acute kidney injury. Their model achieved AUROCs of 0.910, 0.890, and 0.700 for respiratory failure, circulatory failure, and acute kidney injury, respectively, underscoring the value of sequential modeling for dynamic clinical decision support.

Findings from systematic reviews and meta-analyses further support the general advantage of ML models over conventional scoring systems, largely due to their improved predictive capabilities. A growing body of evidence indicates that AI models developed using ensemble learning or deep learning algorithms demonstrate significantly greater discriminative performance—commonly measured by the AUROC—for predicting key clinical outcomes, such as AP severity, organ failure, and mortality, than traditional scoring methods do. For example, a recent meta-analysis reported an average AUROC of 0.88 for ML models, compared with the typical range of 0.74–0.77 for conventional scores.^165^ A detailed comparison of the technical features and performance metrics of 14 representative data-driven models is presented in Table 2.

**Table 2:** Comparison of representative data-driven models for acute pancreatitis prediction

Study (Year) [Ref.]	Core algorithm/model	Dataset details	Prediction endpoint	Model AUROC	External validation	Clinical implications
**Data-driven statistical models**						
Hong et al.^30^	LR (SABP)	Dev: 700, Ext. Val: 194 (Prospective, China)	SAP	0.872-0.875 (External)	Yes	Early bedside triage aid
Wiese et al.^540^	LR (4 indicators)	89 patients (Single-center, retrospective)	IPN	0.819 (Internal)	No	IPN prediction & treatment guidance
Sun et al.^541^	LR (APSAVE)	Dev: 234, Int. Val: 568 (Single-center, China)	SAP	0.730 (Internal)	No	Early biomarker-based triage
Xiao M et al.^171^	Logistic regression & Nomogram	Dev: 431 SAP patients (Single-center, China)	Risk of SAP complicated by SPH	Training: 0.95, Validation: 0.98	No	Early identification & intervention for SAP-SPH, improved prognosis
**Machine learning (Ensemble)**						
Thapa et al.^166^	XGBoost (EHR)	61,894 (Multi-center US, retrospective)	SAP requiring ICU	0.921 (CV)	No	ICU resource optimization
Kui et al.^172^	XGBoost (EASY-APP)	Dev: 1184, Ext. Val: 3543 (International multi-center)	SAP	0.810 (External)	Yes	Generalizable decision support
Hong et al.^164^	RForest (16 features)	648 (Single-center, China)	POF/SAP	0.960 (Internal)	No	POF prediction & intensive care guidance
Zhou et al.^542^	XGBoost	441 (Single-center, China)	AP Severity	0.906 (Internal)	No	AP severity prediction & early ID
Luo et al.^543^	RForest + Nomogram	631 (Multi-center, China)	Persistent Organ Failure	0.955 (External)	Yes	Early SAP prediction & treatment guidance
Yin et al.^544^	AutoML (GBM)	Dev: 1012, Ext. Val: 212 (Multi-center, China)	SAP	0.945 (External)	Yes	High-risk ID & early intervention
Jiang et al.^167^	XGBoost (Interpretable)	Dev: 1782 (MIMIC, US), Ext. Val: 507 (eICU, US)	In-ICU Mortality	0.89 (External)	Yes	Mortality prediction & resource optimization
Yuan et al.^545^	XGBoost (APCU)	Dev (Internal): 5280, Ext. Val: 180 (China)	ICU Admission (within 48 h)	Internal: 0.950, External: 0.873	Yes	ICU admission prediction & resource optimization
**Machine learning (Deep learning/hybrid)**						
Chen et al.^168^	CNN + Clinical data	Dev: 783, Int. Val: 195 (Single-center, China)	AP Severity	0.920 (Internal)	No	Early SAP prediction & treatment guidance
Luo et al.^169^	MT-GRU (Time series)	13,645 (Single-center, China, CV)	Organ Failure (RF, CF, AKI)	RF:0.910, CF:0.890, AKI:0.700(CV)	No	Real-time risk prediction & intervention aid

**Notes:** Model **AUROC** (area under the receiver operating characteristic curve) indicates the model’s discriminative ability; values closer to 1 signify better performance. “(Internal)” or “(CV)” denotes the AUROC derived from internal validation (e.g., train/test split, cross-validation); external validation indicates that the study validated the model via datasets independent of model development (e.g., different time periods, centers, or public databases), generally implying better generalizability. Since the main text does not cover the various predictive models mentioned in the table, their details are provided separately herein

*3D* three-dimensional, *AKI* acute kidney injury, *AP* acute pancreatitis, *APCU* acute pancreatitis critical care unit admission prediction model, *APSAVE* acute pancreatitis stratification using venous blood, *AUROC* area under the receiver operating characteristic curve, *AutoML* automated machine learning, *BISAP* bedside index for severity in acute pancreatitis, *CF* circulatory failure, *CNN* convolutional neural network, *CT* computed tomography, *CV* cross-validation, *Dev* development dataset, *EASY-APP* early assessment score for severity in acute pancreatitis, *EHR* electronic health record, *eICU* eICU collaborative research database, *Ext. Val* external validation dataset/cohort, *GBM* gradient boosting machine, *GRU* gated recurrent unit, *ICU* intensive care unit, *ID* identification, *Int. Val* internal validation dataset/cohort, *IPN* infected pancreatic necrosis, *LR* logistic regression, *MIMIC* medical information mart for intensive care, *MT-GRU* multi-task and time-aware gated recurrent unit, *POF* persistent organ failure, *RF* respiratory failure, *RF* random forest, *SABP* score based on age, bun, and pleural effusion, *SAP* severe acute pancreatitis, *SPH* sinistral portal hypertension, *XGBoost / XGB* extreme gradient boosting

#### Key predictive factors and data processing

The application of AI models in AP extends beyond improving predictive accuracy; it also contributes meaningfully to identifying key prognostic factors within the disease’s pathophysiology. By examining model mechanisms and applying interpretability techniques, researchers have consistently highlighted several variables that are strongly associated with AP severity and adverse outcomes, thereby allowing for more precise confirmation and quantification of their prognostic value. For example, using Shapley Additive Explanations, Jiang et al.^167^ reported that the dynamic trajectory of BUN contributed approximately 35% to the model’s prediction of ability to predict ICU mortality—substantially more than the static admission BUN value. This finding not only supports the clinical relevance of continuous BUN monitoring but also suggests that timely surveillance and management of renal function and volume status may offer a therapeutic window for improving outcomes. In addition to BUN dynamics, other variables frequently identified as significant predictors include CRP, serum creatinine, and albumin levels, the duration of SIRS, patient age, blood glucose levels, and imaging-based indicators such as pleural effusion and CT-derived assessments such as the MCTSI.^30,170^ A recent study by Xiao et al. identified four independent risk factors—male sex, Modified CTSI score, white blood cell count, and portal venous lesions—for sinistral portal hypertension in patients with SAP. Their predictive nomogram demonstrated excellent discriminative performance, with an area under the curve ranging from 0.95 to 0.98, and may serve as a useful clinical tool to help prevent potentially fatal bleeding complications.^171^ In addition to traditional risk prediction tasks, large language models, as emerging AI tools, have begun to demonstrate unique potential in clinical decision support. The results revealed that the model achieved 82–85% accuracy in distinguishing between MAP and SAP. Moreover, its recommendations for critical therapeutic decisions—including the timing of nutritional support, the use of antibiotics, and indications for necrotic tissue drainage—demonstrated a concordance rate of 78–85% with guideline-based recommendations. This development reflects a shift in AI applications from purely predictive modeling toward assisting clinicians in delivering standardized, evidence-based care.

Importantly, rigorous data preprocessing—such as appropriate handling of missing values, standardization of continuous variables, and thoughtful feature engineering—is essential for the development of reliable AI models. These steps not only preserve the integrity of the input data and improve the predictive performance but also support the validity of downstream interpretability analyses. Moreover, given the inherently opaque nature of many high-performing ML models, particularly deep learning architectures, the integration of Explainable AI techniques is gaining increasing attention. Model-agnostic interpretability methods, such as Shapley Additive Explanations and Local Interpretable Model-agnostic Explanations, help to unpack these complex models by quantifying the influence of individual input features on either specific predictions or overall model behavior. In doing so, they translate complex algorithmic reasoning into clinically meaningful insights.^172^ The use of Explainable AI not only enhances transparency but also clarifies how key predictors contribute to model decisions, thereby supporting clinician confidence and enabling more informed application of AI-driven tools in practice.

#### Technical challenges and future directions

Despite the considerable promise shown by AI in risk stratification and prognostic prediction for AP, translating these research advances into broadly adopted clinical tools remains challenging. A major obstacle is the widespread lack of robust external validation for existing models. As noted by Critelli et al., ^164,173^ among 30 recent studies on ML models for AP, only a minority underwent rigorous, independent external validation. Indeed, over 80% of these studies were validated solely on internal datasets, limiting their generalizability and applicability across diverse clinical settings. Another critical issue concerns model interpretability. Many high-performing models, especially those employing complex algorithms such as deep learning, function as “black boxes”, with decision-making processes that are not easily understood. This opacity can hinder clinician trust, as medical decisions require clear and justifiable reasoning. In addition, integrating AI models into existing clinical workflows and demonstrating their real-world utility represent substantial hurdles. On a technical level, compatibility with heterogeneous electronic health record systems often requires intensive data mapping, interface development, and system harmonization. Models relying on real-time data input (e.g., continuous vital signs) can also strain existing IT infrastructure. In addition to technical challenges, clinical adoption depends on careful planning, comprehensive training, and the thoughtful use of explainable AI strategies to enhance transparency and clinician engagement. Methodological rigor is equally important; some recent studies have not fully followed international reporting standards (e.g., TRIPOD-AI), which may introduce bias and compromise reproducibility.^173,174^ Finally, the absence of well-established regulatory frameworks—alongside valid concerns regarding algorithmic bias, data privacy, and accountability—presents significant ethical and legal barriers to the broader implementation of AI in clinical settings. To promote the clinical translation of AI in AP, future work should focus on systematically addressing these challenges. This includes conducting large-scale, multi-center prospective validation studies that comply with established guidelines and assess clinically meaningful outcomes, such as reduced mortality, complications, or hospital stay.^173^ Technological progress should also emphasize the development of inherently interpretable model architectures, balancing performance with transparency to foster clinician trust. Additionally, the potential of large language models to serve as “clinical copilots” warrants thoughtful consideration. Future research should not only aim to validate their predictive capabilities but also explore their broader applications—such as integrating patient data, aligning clinical decisions with up-to-date guideline recommendations in real time, and generating personalized diagnostic and therapeutic suggestions. Moreover, the development of robust validation protocols and regulatory frameworks will be essential to ensure their safety, reliability, and responsible implementation. Ultimately, realizing the full potential of AI in AP management requires not only rigorous clinical validation but also a strong ethical foundation. This involves building regulatory frameworks that ensure data privacy, fairness, and accountability. Through such integrated efforts, AI may become a trustworthy and practical tool to support clinicians and improve outcomes for patients with AP.

In the future, advancing the prediction of AP severity will likely require breakthroughs in several key areas: the development of dynamic prediction models grounded in disease pathophysiology; the integration of multi-omics data with AI-driven analytics; the establishment of predictive–intervention linkage systems that couple risk stratification with tailored therapies (such as targeted anti-inflammatory treatment); and the implementation of multicenter clinical studies to evaluate the real-world impact of novel predictive tools on patient outcomes. Nevertheless, regardless of how prediction tools evolve, the clinical judgment of experienced physicians remains central—especially in recognizing early warning signs such as SIRS and interpreting dynamic physiological changes, where clinical intuition remains irreplaceable. Recent guidelines advocate for a “three-dimensional assessment strategy”, combining clinical manifestations (e.g., persistent abdominal pain, dyspnea), laboratory indicators (e.g., changes in BUN and creatinine), and scoring systems (such as the BISAP or Glasgow-Imrie scores) for a more holistic evaluation.^92,125^ This integrated approach improves predictive accuracy—particularly for identifying moderate to severe cases—while maintaining clinical feasibility, reflecting the current direction of AP severity assessment. In addition, several critical risk factors must be considered comprehensively. Key warning signs include the presence of SIRS,^175^ evidence of hypovolemia (such as elevated BUN^176^ and hematocrit^177^), obesity,^178^ pleural effusion and/or pulmonary infiltrates,^179^ and altered mental status.^180^ Therefore, improving diagnostic accuracy and risk stratification in AP necessitates the formation of interdisciplinary research teams—drawing on expertise from pancreatic disease specialists, imaging and AI, biomarker development, epidemiology, and bioinformatics. Such collaborative platforms are essential for creating next-generation predictive models tailored to diverse clinical populations.^181,182^

## Pathological features and mechanisms of multilevel regulation

The pancreas is the only organ in the body that comprises both exocrine and endocrine components. The exocrine portion accounts for approximately 90% of the pancreas and includes the acini, central acinus, and ductal structures. In contrast, the endocrine component, which is made up primarily of the islets of Langerhans, comprises approximately 1–2% of the pancreatic tissue. The remaining portion consists of blood vessels, lymphatic vessels, nerves, and fibrous connective tissue stroma.^183^ AP is predominantly associated with dysfunction of the exocrine pancreas. The pancreatic acinus is a highly polarized structure composed of a single layer of pyramidal acinar cells arranged concentrically around a central lumen. The apical region of these cells is densely packed with eosinophilic zymogen granules (ZGs), whereas the basolateral region is basophilic due to the presence of nuclei and abundant rough endoplasmic reticulum (ER), where substantial protein synthesis occurs. Inactive digestive enzymes are packaged into condensing vacuoles by the Golgi apparatus and stored in ZGs at the apical pole of acinar cells.^184^ Upon secretion into the pancreatic ducts and subsequent entry into the intestinal lumen, these inactive enzymes are activated by enterokinase, enabling the digestion of proteins, carbohydrates, and lipids. Simultaneously, acinar cells secrete a sodium chloride-rich fluid that facilitates enzyme transport.^185^ The ductal structures form the pancreatic ductal system and express carbonic anhydrase, which produces bicarbonate and water. This fluid not only propels digestive enzymes into the duodenum but also neutralizes gastric acid.^186^ The coordinated synthesis, processing, and secretion of digestive enzymes, along with fluid and electrolyte secretion, are essential for preserving pancreatic integrity and function.^186^

### Lysosomal cathepsin dysfunction

The premature activation of digestive enzymes—particularly trypsinogen—within the pancreas is a central mechanism in the pathogenesis of AP. Evidence suggests that aberrant activation of lysosomal cathepsins, which play a key regulatory role in trypsinogen activation, may represent an early pathological event in acinar cell injury during the onset of AP. Lysosomes consist of an acidic lumen enclosed by a phospholipid bilayer membrane. The lumen contains approximately 50 types of hydrolases—including proteases, lipases, nucleases, glycosidases, phospholipases, phosphatases, and sulfatases—which function optimally at low pH. The inner surface of the lysosomal membrane is lined with a dense glycocalyx that protects the membrane from degradation by lysosomal hydrolases and helps maintain the compartmentalization of the acidic environment. This compartmentalization is critical, as rupture of the lysosomal membrane may result in the release of multiple hydrolytic enzymes into the cytoplasm, potentially leading to premature trypsinogen activation and the initiation of AP.

#### Cathepsin B

Cathepsin B (CTSB) plays a key role in early trypsinogen activation during the onset of pancreatitis. Administration of the CTSB inhibitor CA-074me prior to pancreatitis induction in a rat model significantly reduced intrapancreatic trypsinogen activation.^187^ Although CA-074Me has been shown to significantly inhibit the enzymatic cascade, its clinical application in patients with AP remains limited. This is largely due to a mismatch between its optimal window of intervention—ideally before or within one hour of disease onset—and real-world clinical scenarios. In practice, patients with AP are typically admitted after the onset of significant symptoms, at which time the optimal opportunity for early intervention is often missed. However, a subsequent study revealed that CTSB contributes not only to the activation of trypsinogen within acinar cells but also within infiltrating macrophages, thereby exacerbating pancreatitis through NF-κB nuclear translocation and the production of inflammatory mediators.^188^ These findings suggest that CTSB inhibitors may act on both acinar cells and macrophage-driven inflammation and that dual-targeted strategies focusing on CTSB and its downstream signaling (e.g., NF-κB) may offer a more comprehensive approach to treatment. For example, salidroside has been shown to alleviate pulmonary fibrosis in rats by reducing the expression of CTSB and NF-κB p65,^189^ providing a potential therapeutic avenue for AP. Additionally, the co-localization or fusion of lysosomal CTSB with trypsinogen in ZGs appears to be a prerequisite for AP development.^190^ One study further explored the downstream consequences of this co-localization and demonstrated for the first time that cytoplasmic CTSB, rather than trypsin, initiates acinar cell death in AP. During disease progression, the contents of co-localized organelles—including lysosomal enzymes and digestive proenzymes—are released into the cytoplasm, where CTSB activates cell death pathways. These findings suggest that the role of trypsin may be primarily to facilitate the release of CTSB into the cytosol, which helps explain why the inhibition of either CTSB or trypsin can prevent acinar cell death.^191^ The hypothesis that cytoplasmic CTSB triggers apoptosis was further supported by findings showing that CTSB promotes caspase–1–mediated pyroptosis and exacerbates AP via NLRP3 inflammasome activation.^192^ Accordingly, strategies that block CTSB activation and release can protect acinar cells and limit tissue injury, while concurrently targeting the NLRP3 inflammasome may enhance these protective effects, suggesting a promising dual-targeted therapeutic approach. For example, lycopene may exert therapeutic effects by inhibiting the CTSB/NLRP3 signaling pathway,^193^ further supporting the rationale for combined intervention in AP.

#### Cathepsin L

CTSB activates trypsinogen, whereas cathepsin L (CTSL) inactivates it.^194^ Some researchers have proposed that the pathogenesis of AP may be associated with the balance between CTSB and CTSL activity. However, in mice lacking both CTSB and CTSL, only trypsin activity was altered, with no significant impact on disease severity or inflammatory responses following caerulein-induced AP.^195^ Moreover, in CTSL-deficient mice, both trypsin activity and pancreatic apoptosis were increased, yet disease severity was paradoxically reduced.^194^ These findings suggest that CTSL primarily antagonizes CTSB-mediated trypsin activation but does not directly influence AP outcomes. Therefore, while CTSB inhibition appears to reduce trypsin activation and mitigate disease severity, modulating CTSL activity may not confer similar therapeutic benefits.

#### Cathepsin D

Cathepsin D (CTSD), an aspartic protease, is expressed in both pancreatic acinar cells and inflammatory cells. Recent studies have shown that CTSD regulates AP by directly activating CTSB rather than trypsin. Although CTSD deficiency results in minimal and short-lived effects during the early, acinar cell–dependent phase of AP, its impact becomes more pronounced during the later, inflammation-driven phase.^196^ These findings suggest that CTSD contributes to AP progression not only through its actions in acinar cells but also via its role in inflammatory cells. Given its involvement in inflammatory responses, CTD-002—a potent small-molecule inhibitor that selectively targets the extracellular domain of CTSD—may offer therapeutic potential for mitigating inflammation in the later stages of pancreatitis.^197^

#### Cathepsin C

Cathepsin C (CTSC) is a widely expressed exocysteine protease that regulates and activates various lysosomal enzymes, including CTSB and CTSD. CTSC deficiency has been shown to attenuate the severity of pancreatitis, not by inhibiting proenzyme activation in acinar cells but by reducing neutrophil infiltration into the pancreas.^198^ B22, a novel indolinone derivative, inhibits CTSC activity and has been reported to reduce both inflammation and cytokine levels,^199^ highlighting CTSC as a potential therapeutic target for limiting disease progression in pancreatitis.

In summary, CTSB, CTSL, CTSD, and CTSC each play distinct and important roles in the pathogenesis of AP. Among these factors, CTSB is considered a principal driver of disease initiation, whereas CTSD and CTSC contribute to the propagation of inflammation during AP progression. Currently, the colocalization of lysosomes and ZGs represents one of the earliest pathological events in acinar cells during AP. However, Zierke et al., using a Rab7 knockout mouse model—Rab7 being a key GTPase involved in lysosome–vesicle fusion—observed that trypsinogen activation still occurred and pancreatic injury was not substantially delayed, even when lysosome–zymogen granule fusion was disrupted.^190^ These results indicate that the “co-localization hypothesis” alone may not fully explain the morphological alterations of ZGs or the activation of zymogens.

### Calcium signaling

**Fig. 3:**
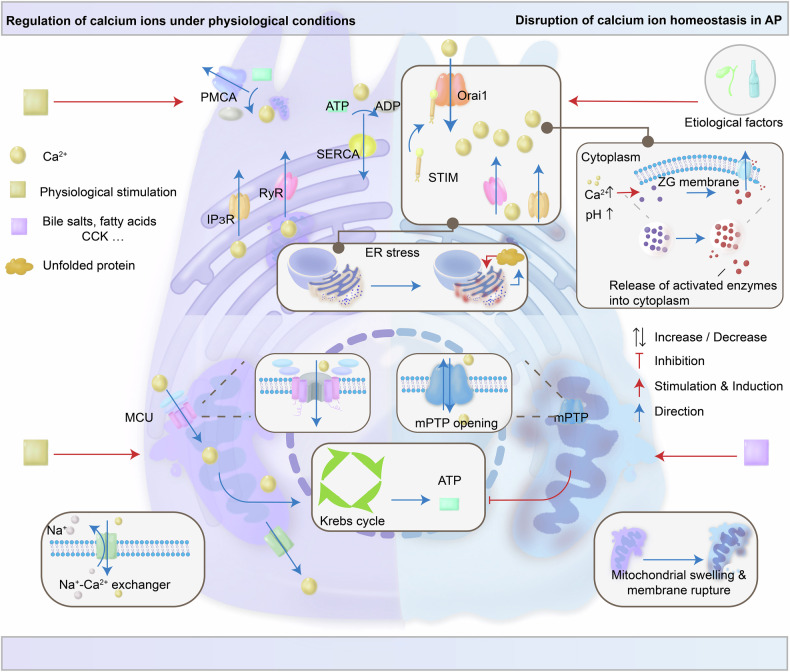
Physiological and pathological calcium regulatory mechanisms in pancreatic acinar cells. The ER and mitochondria coordinate to maintain intracellular Ca²⁺ homeostasis in acinar cells. Under physiological stimulation (left panel), the activation of IP₃Rs and RyRs mediates Ca²⁺ release from the ER into the cytosol. Upon ER Ca²⁺ store depletion, STIM senses the change and binds to Orai1 on the plasma membrane, initiating store-operated calcium entry to replenish ER Ca²⁺. Mitochondria take up cytosolic Ca²⁺ through MCU channels to support metabolic regulation and ATP synthesis, whereas Na⁺/Ca²⁺ exchangers maintain mitochondrial Ca²⁺ homeostasis. ATP generated via the Krebs cycle provides the energy required for SERCA and PMCA pump function. In acute pancreatitis (right panel), pathological stimuli, such as bile salts, alcohol metabolites, and fatty acids, disrupt intracellular Ca²⁺ homeostasis. Aberrant activation of IP₃Rs and RyRs leads to excessive Ca²⁺ release from the ER, whereas dysregulation of the STIM1-Orai1 axis exacerbates cytosolic Ca²⁺ overload. ER stress subsequently activates the unfolded protein response and impairs cellular functions. Simultaneously, mitochondrial uptake of excess Ca²⁺ triggers mPTP opening, resulting in mitochondrial swelling, membrane rupture, and impaired ATP production. Elevated cytosolic Ca²⁺ and increased pH promote premature activation of zymogen granules, leading to the release of digestive enzymes into the cytosol. AP acute pancreatitis, ATP adenosine triphosphate, ER endoplasmic reticulum, IP₃R inositol 1,4,5-trisphosphate receptor, MCU mitochondrial calcium uniporter, mPTP mitochondrial permeability transition pore, PMCA plasma membrane Ca²⁺-ATPase, RyR ryanodine receptor, SERCA sarco/endoplasmic reticulum Ca²⁺-ATPase, STIM stromal interaction molecule, ZG zymogen granule

Ca²⁺, a ubiquitous intracellular second messenger, is primarily stored in the endoplasmic/sarcoplasmic reticulum and mitochondria, where it plays a critical role in regulating pancreatic fluid and enzyme secretion.^200^ Studies have shown that excessive intracellular Ca²⁺ levels—beyond the normal temporal and spatial range—can lead to acinar cell secretory dysfunction, premature activation of pancreatic enzymes, microcirculatory impairment, oxidative stress, and mitochondrial dysfunction (Fig. 3). These disturbances not only are key contributors to the pathogenesis of AP but are also considered among the earliest pathological events in acinar cells during AP onset.^201^

#### Endoplasmic reticulum and calcium signaling

The release of Ca²⁺ stored in the ER is a key contributor to Ca²⁺ overload in acinar cells. Two types of Ca²⁺ release channels exist on the ER membrane: inositol 1,4,5-trisphosphate receptors (IP_3_Rs) and ryanodine receptors (RyRs). IP_3_Rs mediate the Ca²⁺ spike response in the apical cytoplasm triggered by physiological concentrations of the neurotransmitter acetylcholine, whereas RyRs are activated by nicotinic acid adenine dinucleotide phosphate and cyclic ADP-ribose in response to physiological levels of cholecystokinin.^202^ Under normal conditions, acetylcholine and cholecystokinin within physiological ranges induce Ca²⁺ exocytosis in the granular area at the acinar tip and extracellular trypsinogen secretion via IP_3_R or nicotinic acid adenine dinucleotide phosphate without activating trypsinogen stored in granules.^203,204^ However, pathological stimuli such as bile acids and alcohol, beyond certain concentrations, provoke massive Ca²⁺ release from the ER through IP_3_Rs and RyRs. Sustained cytoplasmic Ca²⁺ elevation destabilizes ZGs, which also serve as Ca²⁺ stores. ZGs can release Ca²⁺ and allow potassium influx through IP_3_R- and cyclic ADP-ribose-gated channels, increasing the pH within these granules. This process triggers premature activation of digestive enzymes and ZG vacuolization,^205^ followed by leakage of activated enzymes into the cytoplasm, resulting in cytoskeletal disruption and plasma membrane damage.^203^ Moreover, disruption of ER Ca²⁺ homeostasis may impair ER protein folding, inducing ER stress and unfolded protein responses that can lead to apoptosis.^201^ The ER membrane also contains transient receptor potential channel 1 and stromal interaction molecules, which sense ER Ca²⁺ depletion. Upon sensing decreased ER Ca²⁺, stromal interaction molecules translocate to the plasma membrane to colocalize with Orai1 channels, activating store-operated Ca²⁺ influx to replenish ER Ca²⁺ stores.^202^ Notably, Orai1 is expressed on both the acinar cell membrane and the luminal plasma membrane of vascular endothelial cells. Inhibiting Orai1 activity reduces stromal interaction molecule-dependent extracellular Ca²⁺ influx induced by bile acids or alcohol, thereby preventing pancreatic duct secretion damage and potentially improving AP prognosis.^206^ For example, the calcium channel inhibitor CM4620 (Auxora, CalciMedica) selectively blocks the Orai1 channel and inhibits calcium influx, thereby significantly reducing acinar cell injury and inflammation in animal models. Phase II clinical trials have also demonstrated that CM4620 can provide symptomatic relief and reduce the incidence of SIRS.^207,208^ Ultrastructural studies have shown that most of the ER surrounds the nucleus, with extensions reaching the apical granular region near the plasma membrane.^200^ Given the critical role of Ca²⁺ overload in acinar cells, targeting Ca²⁺ signaling pathways may offer therapeutic benefits for AP. Specifically, the inhibition of IP_3_R or RyR channels could prevent excessive Ca²⁺ release, reducing digestive enzyme activation and subsequent cell injury. For example, methylxanthines—particularly caffeine—suppress IP₃R-mediated Ca²⁺ release, thereby reducing toxic intracellular Ca²⁺ elevations and alleviating the severity of AP.^209^ Safety studies have also reported favorable profiles for caffeine use in this context.^210^ In addition, urolithin A has shown therapeutic potential by modulating the IP₃R–VDAC1 channel and inhibiting programmed necrosis in SAP.^211^ Although IP₃R/RyR inhibitors and Orai1 blockers have demonstrated considerable therapeutic promise in AP, systematic evaluations of their mechanisms of action, pharmacokinetics, and safety profiles are still lacking, which hampers their clinical translation. Moreover, a dual-channel therapeutic strategy—combining an IP₃R inhibitor with an Orai1 blocker—may be worth exploring, as it targets both calcium release and influx, potentially increasing treatment efficacy.

#### Mitochondria and calcium signaling

Mitochondria, another important Ca²⁺ reservoir, contribute to maintaining cytosolic Ca²⁺ homeostasis in pancreatic acinar cells.^202^ They are distributed around secretory granules, beneath the plasma membrane, and near the nucleus, reflecting their specialized physiological roles.^200^ Upon physiological stimulation, an increase in cytosolic Ca²⁺ is sensed by mitochondria, which absorb Ca²⁺ via the mitochondrial calcium uniporter and mitochondrial calcium uptake 1. This uptake prevents the Ca²⁺ signal from spreading to the basal lateral region of the acinar cell, where the nucleus resides. The mitochondria surrounding the granules act as a Ca²⁺ buffering barrier, regulating Ca²⁺ uptake termination and removing Ca²⁺ through the mitochondrial Na⁺-Ca²⁺ exchanger. Mitochondrial Ca²⁺ influx activates the Krebs cycle and drives ATP production, supplying the energy needed for Ca²⁺ reabsorption by the ER and extrusion by sarco/ER Ca²⁺-ATPase and plasma membrane Ca²⁺-ATPase pumps.^202^ Pathologic stimuli such as bile salts, fatty acids, and cholecystokinin or their analogs induce depolarization of the mitochondrial inner membrane, decreasing the membrane potential and impairing ATP production. This dysfunction inhibits Ca²⁺ reuptake by mitochondria near the granules, reducing their ability to buffer local Ca²⁺ increases near the apical cell surface. Consequently, Ca²⁺ signals spread throughout the entire acinar cell. ATP depletion further diminishes sarco/ER Ca²⁺-ATPase and plasma membrane Ca²⁺-ATPase pump activity, impairing Ca²⁺ clearance from the cytosol and contributing to intracellular Ca²⁺ overload. Prolonged mitochondrial Ca²⁺ overload triggers opening of the mitochondrial permeability transition pore (mPTP), causing increased membrane permeability, loss of membrane potential, mitochondrial swelling, membrane rupture, and further ATP depletion.^212^ This cascade, involving ATP deficiency, Ca²⁺ overload, and protease activation, leads to acinar cell necrosis and exacerbates the inflammatory response.^201^ Targeting mitochondrial dysfunction offers a potential therapeutic approach for alleviating Ca²⁺ overload and reducing acinar cell necrosis in AP. Strategies aimed at stabilizing the mitochondrial membrane potential, preventing swelling, and enhancing ATP production or mitochondrial Ca²⁺ uptake via the mitochondrial calcium uniporter may be beneficial. For example, oleuropein activates mitochondrial Ca²⁺ uptake and increases energy metabolism,^213^ potentially improving mitochondrial function and reducing cellular injury in AP. To prevent mitochondrial calcium overload, Ru360—an inhibitor of mitochondrial calcium uniporters—has demonstrated protective effects on pancreatic tissue in both in vitro and in vivo preclinical models.^214,215^ Moreover, a novel cyclosporine A derivative has shown clear tissue-protective effects in animal models of AP by inhibiting the mPTP.^216^ However, clinical data on its use in pancreatitis remain limited. In addition, most proposed therapeutic strategies are still at the stage of early basic research, and systematic evaluations of their target specificity, toxicity, and clinical translational potential are lacking. Future studies may explore the role of mitochondria–calcium signaling crosstalk as a synergistic regulatory mechanism in AP.

In summary, pathological stimulation triggers excessive Ca²⁺ release from the ER and disrupts mitochondrial function, thereby impairing Ca²⁺ uptake and reducing ATP production. These alterations contribute to intracellular Ca²⁺ overload, elevated intracellular pH, premature activation of digestive enzymes, and acinar cell injury, which are early events in the pathogenesis of AP (Fig. 3). Therefore, restoring ER and mitochondrial function to stabilize intracellular Ca²⁺ signaling may represent a promising therapeutic approach for mitigating inflammation in AP.

### Autophagy and its interaction with organelles

Autophagy is also believed to be an early event in the development of AP. As a highly conserved cellular process, autophagy plays a critical role in maintaining cellular homeostasis during nutrient deprivation.^217,218^ In this process, damaged, dysfunctional, or redundant organelles, as well as long-lived proteins and lipids within the cytoplasm, are delivered to lysosomes and degraded by lysosomal hydrolases. Autophagy can be categorized into three types on the basis of the mode of substrate delivery to lysosomes: macroautophagy, microautophagy, and chaperone-mediated autophagy.^218^ Macroautophagy appears to be the predominant form detected in the normal exocrine pancreas and during pancreatitis; hereafter, it is simply referred to as “autophagy”. Depending on the degradation mechanism, autophagy can be either non-selective or selective. Non-selective autophagy, typically induced by nutrient deprivation, randomly engulfs cytoplasmic components near the site of autophagosome formation. In contrast, selective autophagy specifically targets substrates recognized by autophagy proteins, such as those involved in ER phagocytosis.^219^

**Fig. 4:**
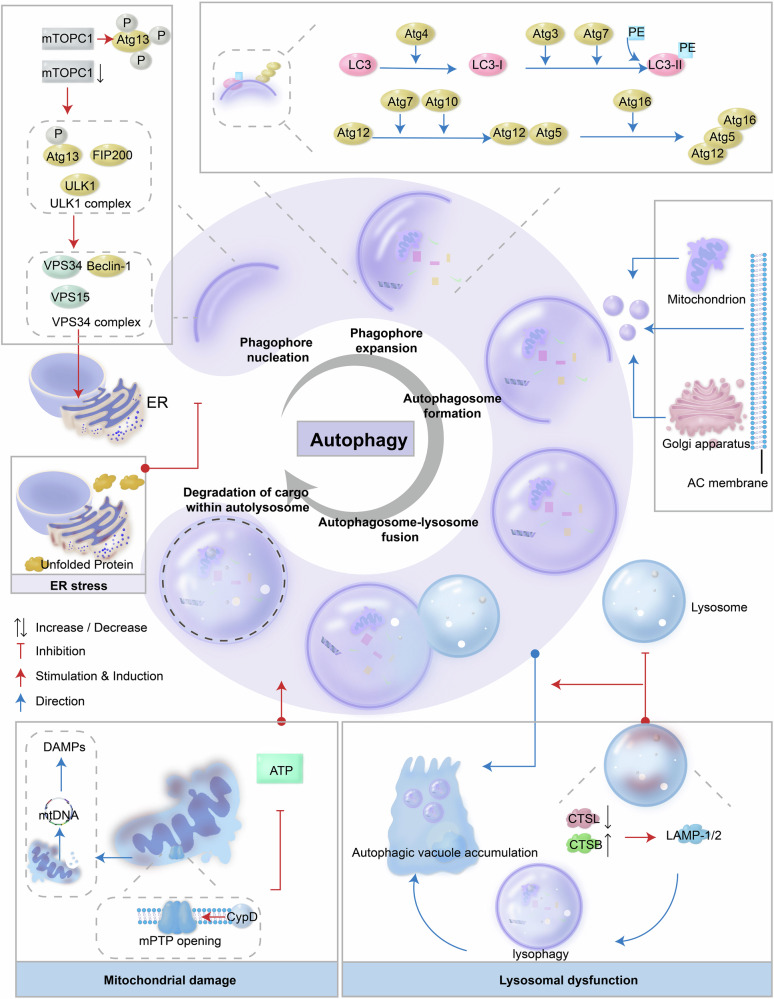
Regulatory mechanisms of autophagy in acute pancreatitis. Autophagy is tightly regulated by multiple signaling pathways and organelle functions. Inhibition of mTORC1 activates the ULK1 complex (composed of ULK1, Atg13, and FIP200), initiating autophagosome formation. The VPS34 complex subsequently mediates the nucleation of the phagophore. Autophagosome elongation relies on two ubiquitin-like conjugation systems: the ATG12–ATG5–ATG16L1 complex and the LC3 lipidation pathway. Membrane sources derived from the ER, mitochondria, and Golgi apparatus contribute to autophagosome biogenesis. Under physiological conditions, autophagosomes fuse with lysosomes to form autolysosomes, enabling the degradation and recycling of cellular components. In AP, however, dysregulation or aberrant activation of lysosomal cathepsins (e.g., CTSL and CTSB) leads to degradation of LAMP-1/2, impairing autophagosome–lysosome fusion and resulting in autophagosome accumulation and cytoplasmic vacuolization. Damaged lysosomes may also be selectively removed via lysophagy. CypD-mediated mitochondrial depolarization and opening of the mPTP reduce ATP production, further inhibiting autophagy. Additionally, damaged mitochondria release mtDNA into the cytosol; acting as a DAMP, mtDNA triggers inflammatory signaling cascades, contributing to pancreatic necrosis and immune activation. ER stress, which is characterized by the accumulation of unfolded or misfolded proteins, further impairs autophagosome membrane formation and exacerbates cellular dysfunction. Taken together, these organelle dysfunctions disrupt autophagic flux, representing a key pathogenic mechanism in AP. AC acinar cell, ATP adenosine triphosphate, Atg autophagy-related gene/protein (e.g., Atg3, Atg4, Atg5, Atg7, Atg10, Atg12, Atg13, and Atg16), CTSB cathepsin B, CTSL cathepsin L, CypD cyclophilin D, DAMPs damage-associated molecular patterns, ER endoplasmic reticulum, FIP200 focal adhesion kinase family interacting protein of 200 kDa, LAMP-1/2 lysosome-associated membrane protein 1/2, LC3-I/LC3-II microtubule-associated protein 1 light chain 3-I/II, mPTP mitochondrial permeability transition pore, mTORC1 mechanistic target of rapamycin complex 1, mtDNA mitochondrial DNA, ULK1 unc-51 like autophagy activating kinase 1, VPS15 vacuolar protein sorting 15, VPS34 vacuolar protein sorting 34

Recent studies have identified a persistent and prominent feature of experimental pancreatitis in humans and animals: the accumulation of large vacuoles in acinar cells. These vacuoles mainly consist of autophagosomes and lysosomes, accompanied by increased levels of the LC3-II and p62 proteins and decreased levels of the LAMP-2 protein.^219,220^ Research indicates that autophagy is activated while lysosomal degradation is impaired in acinar cells during AP, contributing to vacuole accumulation. In GFP-LC3 transgenic mice overexpressing LC3, which are used to manipulate autophagy and model pancreatitis, the number and size of autophagic vacuoles in the pancreas are significantly greater than those in wild-type mice, which is correlated with pancreatitis severity. Notably, these transgenic mice presented minimal or no changes in autophagy in the liver, lung, or spleen,^221^ suggesting that autophagy disruption is closely linked to AP progression. Organelles such as the ER, mitochondria, and lysosomes in acinar cells play essential roles in autophagy initiation and regulation (Fig. 4); dysfunction of these organelles impairs autophagic flux and promotes pancreatitis development.

#### Lysosomes and autophagy

Lysosomes play a direct role in autophagy. Recent studies have identified three mechanisms by which lysosomes contribute to autophagic dysfunction in AP: impaired fusion between autophagosomes and lysosomes, abnormal lysosomal cathepsin activity, and insufficient lysosomal biogenesis.^219^ LAMPs are highly glycosylated transmembrane proteins that constitute approximately 70% of lysosomal membrane proteins. Their heavily glycosylated structure and stable hydrolase complexes protect lysosomes from degradation by acid hydrolases, making the integrity and degradative capacity of lysosomes largely dependent on LAMP-1 and LAMP-2.^219,222^ However, Gukovskaya et al.^217^ reported LAMP degradation in four different rat and mouse models of nonalcoholic pancreatitis, further demonstrating that this process is mediated by lysosomal cathepsins. One possible mechanism involves abnormal cathepsin maturation, which alters its interaction and localization within lysosomes during pancreatitis, enabling cathepsins to hydrolyze LAMP proteins. Notably, LAMP proteins, especially LAMP-2, are essential for lysosome‒autophagosome fusion. In mouse embryonic fibroblasts deficient in LAMP-2, this fusion is impaired but can be restored by exogenous LAMP-2a administration.^222,223^ The degradation deficiency caused by LAMP impairment results in the accumulation of autophagosomes containing partially degraded cargo.^224,225^ In LAMP-2-deficient mice, lysosomal dysfunction leads to acinar cell vacuolation and spontaneous pancreatitis characterized by trypsinogen activation and acinar cell death.^226^ Therefore, targeting LAMP-1 and LAMP-2 to restore lysosomal integrity and function represents a promising therapeutic strategy for AP. Furthermore, during pancreatitis, acinar cells exhibit not only autophagosome accumulation but also increased co-localization of lysosomal markers with LC3-II,^227^ indicating that while fusion between autophagosomes and lysosomes is not completely blocked, it may be partially impaired.

Impaired autophagy is also linked to an imbalance between the lysosomal proteases CTSL and CTSB. Specifically, enhanced CTSB-mediated activation of trypsinogen to trypsin, combined with inefficient degradation of trypsin and trypsinogen due to immature CTSL, may contribute to intrapancreatic trypsin accumulation and the progression of pancreatitis,^228^ as discussed in the cathepsin dysfunction section. Therapeutic strategies aimed at restoring the CTSB–CTSL balance or promoting CTSL maturation may help mitigate pathological trypsin activation and reduce pancreatitis severity. Transcription factor EB (TFEB), a key regulator of lysosomal biogenesis and autophagy,^229^ has been increasingly implicated in experimental pancreatitis through its influence on lysosomal abundance and function, thereby modulating autophagic activity.^219^ For example, Wang et al. analyzed GFP-LC3 transgenic mice, acinar cell–specific TFEB knockout mice, TFEB/TFE3 double knockout mice, and human pancreatitis samples. They reported that caerulein administration activated the pancreatic mechanistic target of rapamycin (mTOR), increased phosphorylated TFEB levels, and accelerated TFEB degradation, ultimately reducing TFEB abundance and the number of lysosomes. This lysosomal insufficiency impaired autophagy and contributed to AP pathogenesis. Notably, reduced TFEB nuclear localization is closely associated with human pancreatitis.^230,231^ TFEB deletion exacerbates experimental AP, whereas TFEB overexpression exerts protective effects.^220^ Indeed, adenoviral TFEB overexpression via tail vein injection significantly alleviated alcohol-induced pancreatitis by reducing tissue injury, lowering serum amylase and lipase levels, and partially preserving lysosomal proteins.^231,232^ Moreover, disruption of key autophagy-related genes or proteins, such as ATG5, ATG7, or LAMP2, can impair autophagosome formation, leading to spontaneous pancreatitis or CP.^226,228^ In summary, targeting TFEB—through gene therapy, mTOR inhibition, or enhancement of autophagy-related protein function—may represent a promising therapeutic approach to restore autophagy and alleviate the severity of AP. However, the temporal regulation of lysosome-associated molecules during the progression of AP remains poorly understood. In particular, the stage-specific relationships among LAMP-2 degradation, CTSB/CTSL imbalance, and TFEB dysregulation have yet to be clarified. At present, related research is still at the basic science level, and drug development targeting the lysosome–autophagy system remains in the exploratory phase. Future studies should further investigate the critical role of the lysosome–autophagy axis in the pathophysiology of AP, with the aim of establishing a theoretical foundation for the development of targeted therapeutic interventions. Notably, when autophagy-associated lysosomes become damaged or senescent, they can themselves be degraded through autophagy, serving as a self-protective cellular mechanism.^218^

#### Endoplasmic reticulum stress and autophagy

One of the primary membrane sources for autophagosomes is the ER, and both the initiation and maturation of autophagosomes are closely associated with ER function.^217^ When protein synthesis exceeds the folding capacity of the ER, unfolded or misfolded proteins accumulate, resulting in.^226^ This stress impairs the synthesis of autophagosomal membrane proteins and indirectly disrupts the autophagy process, thereby contributing to the progression of AP. This finding aligns with others’ observations of ER swelling and increased expression of ER stress markers in a mouse model of AP.^233^ Currently, certain compounds have been shown to stabilize the supply function of the ER for autophagic membrane formation. For example, 4-phenylbutyrate can markedly reduce ER stress and help maintain a favorable protein-folding environment in AP models. However, no clinical data are currently available regarding its use in AP.^234–236^ Furthermore, dysregulated Ca²⁺ homeostasis within the ER can promote the transcriptional activation of multiple autophagy-related genes.^219^

Restoring autophagic flux enables the selective degradation of ER fragments containing misfolded proteins and lipids via autolysosomes, a process known as reticulophagy, which can markedly alleviate ER stress and help restore ER function.^237^ For example, in a rat model of high-fat diet-induced pancreatitis, severely impaired autophagy and substantial accumulation of unfolded proteins were observed. When autophagic flux is restored via the use of mTOR inhibitors (e.g., rapamycin) or palmitic acid, ER stress is significantly reduced, thereby preventing HTG-induced AP.^238^ Biczo et al.^239^ reported that ER stress is a downstream pathological consequence of impaired autophagy during AP. In their study, the intraperitoneal administration of trehalose to wild-type mice for two weeks prior to AP induction significantly increased autophagic activity and restored autophagic flux. This improvement in autophagy was associated with reduced ER stress and notable attenuation of AP severity. However, the primary–secondary relationship between ER stress and autophagy remains unclear. The distinct roles of the unfolded protein response in regulating autophagy and determining cell fate have yet to be systematically defined, and whether the modulation of autophagy can alleviate ER stress also remains to be elucidated. Future studies should aim to clarify the stage-specific regulatory patterns of the unfolded protein response pathway during autophagy and identify key signaling nodes that may hold potential for clinical translation.

#### Mitochondrial dysfunction and autophagy

Mitochondria are double-membrane-bound organelles that generate ATP primarily through oxidative phosphorylation. The mitochondrial membrane potential plays a critical role in maintaining mitochondrial function. The mPTP is a non-selective channel that permits the passage of water and solutes with molecular weights less than 1500 Da into the mitochondrial matrix. However, sustained opening of the mPTP can result in membrane depolarization, reduced ATP production, and ultimately, mitochondrial dysfunction.^240^ Cyclophilin D (CypD) is a key regulator of mPTP opening.^241^ CypD-mediated mPTP opening leads to loss of the mitochondrial membrane potential and decreased ATP synthase activity, thereby impairing the autophagy process. For example, L-arginine has been shown to significantly reduce ATP synthase activity and induce mitochondrial Ca²⁺ overload by disrupting mitochondrial function in the pancreas of rats and mice. These effects are attributed to CypD-mediated mitochondrial depolarization.^239^ Furthermore, autophagy impairment—considered a downstream event of mitochondrial dysfunction—has been associated with ER stress and disordered lipid metabolism, eventually contributing to the development of AP. Notably, these pathological changes were alleviated in CypD-knockout mice. Pharmacological strategies that inhibit CypD or stabilize the mitochondrial membrane potential may be promising for preventing or attenuating mitochondrial dysfunction in AP. For example, the mitochondrion-targeted antioxidant mitoquinone mesylate has been shown to reduce pancreatic edema and neutrophil infiltration in a mouse model of pancreatitis.^242^ Although multiple clinical trials have been conducted in human populations,^243,244^ clinical data specific to AP are currently lacking.

Similarly, impaired autophagy may exacerbate mitochondrial dysfunction by preventing the removal of damaged mitochondria, thereby contributing to the progression of AP. The autophagy modulator trehalose has been shown to facilitate the clearance of dysfunctional mitochondria and suppress aberrant trypsinogen activation.^245,246^ Under normal conditions, dysfunctional mitochondria are selectively degraded through a protective process known as mitophagy. During this process, mitochondria harboring damaged mtDNA are sequestered into autophagosomes, where mtDNA is degraded by DNase II.^247^ Disruption of mitophagy may result in the accumulation or cytosolic release of damaged mtDNA, which functions as a damage-associated molecular pattern (DAMP) and activates inflammatory pathways involved in pancreatic necrosis and inflammation.^248^ Additionally, impaired autophagy delays the clearance of dysfunctional mitochondria, further exacerbating mitochondrial dysfunction and reducing ATP production. This reciprocal relationship between autophagy impairment and mitochondrial dysfunction creates a vicious cycle that amplifies AP pathology.^239^ For example, in a hyperlipidemia-induced mouse model of AP, both defective autophagic flux and mitochondrial dysfunction were observed. These defects promoted disease progression by upregulating NLRP3 and IL-1β expression in the pancreas. Treatment with Rhizoma Alismatis Decoction, a traditional Chinese medicine (TCM) formula, attenuated hyperlipidemia-induced AP by inhibiting mitochondrial oxidative stress, enhancing autophagic flux, and promoting mitophagy.^249^

In summary, autophagy, lysosomes, mitochondria, and the ER constitute an interconnected network in which dysfunction in one organelle can influence the others. For example, ER stress can impair autophagy, whereas defective autophagy fails to clear misfolded protein–laden ER, thereby intensifying ER stress. Similarly, mitochondrial dysfunction reduces ATP production, limiting energy availability for autophagy, which in turn impairs mitophagy. This leads to the cytosolic release of mtDNA, triggering inflammatory cascades and worsening mitochondrial damage. Such feedback loops ultimately contribute to the pathogenesis of AP.

### Mode of acinar cell death

**Fig. 5:**
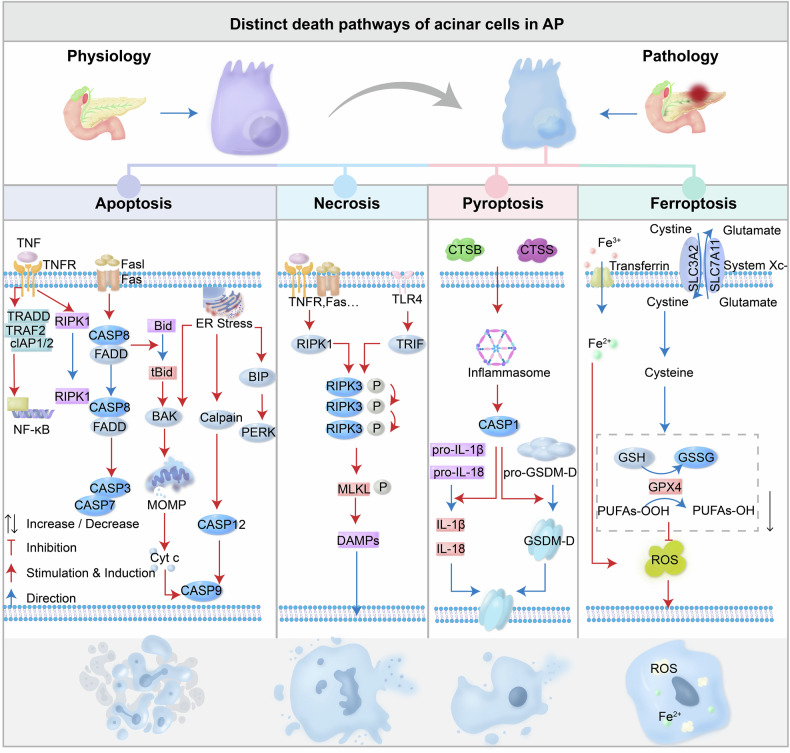
Distinct modes of acinar cell death in acute pancreatitis. This figure provides a structured overview of the main forms of acinar cell death in AP, including apoptosis, necrosis, pyroptosis, and ferroptosis. (1) Apoptosis: Acinar cell apoptosis primarily occurs via the mitochondrial pathway, death receptor pathway, and ER stress pathway. It is characterized by activation of the caspase-8/9/3/7 cascade, increased MOMP, and cytochrome c release. As a non-inflammatory mode of cell death, apoptosis may contribute to protective clearance, particularly in the early or mild stages of AP. (2) Necrosis: This process is mediated by the RIPK3–MLKL signaling axis and typically results in the extensive release of DAMPs, which initiate strong inflammatory cascades. (3) Pyroptosis: Pyroptosis is triggered by inflammasome activation (e.g., NLRP3) and involves caspase-1–mediated cleavage of GSDMD, leading to pore formation in the plasma membrane. This promotes the release of proinflammatory cytokines such as IL-1β and IL-18, substantially amplifying both local and systemic inflammation. (4) Ferroptosis: This oxidative stress-related form of cell death is driven by depletion of the GSH system, loss of GPX4 activity, and abnormal accumulation of lipid ROS, representing a distinct mechanism in AP pathophysiology. AP acute pancreatitis, BAK BCL2-antagonist/killer, BID BH3-interacting domain death agonist, BIP binding immunoglobulin protein, CASP1/3/7/8/9/12 caspase-1/3/7/8/9/12, cIAP1/2 cellular inhibitor of apoptosis protein 1/2, CTSB cathepsin B, CTSS cathepsin S, DAMPs damage-associated molecular patterns, ER endoplasmic reticulum, FADD fas-associated death domain protein, FASL fas ligand, GPX4 glutathione peroxidase 4, GSDMD gasdermin D, GSH glutathione, GSSG glutathione disulfide, IL-1β interleukin-1β, IL-18 interleukin-18, MLKL mixed lineage kinase domain-like protein, MOMP mitochondrial outer membrane permeabilization, NF-κB nuclear factor kappa-light-chain-enhancer of activated B cells, PERK protein kinase R-like endoplasmic reticulum kinase, RIPK1 receptor-interacting serine/threonine-protein kinase 1, RIPK3 receptor-interacting serine/threonine-protein kinase 3, ROS reactive oxygen species, SLC3A2 solute carrier family 3 member 2, SLC7A11 solute carrier family 7 member 11, TLR4 toll-like receptor 4, TNF tumor necrosis factor, TNFR tumor necrosis factor receptor, TRADD TNFR1-associated death domain protein, TRAF2 TNF receptor-associated factor 2

During the course of AP, acinar cells may undergo various forms of cell death in response to different stimuli, including apoptosis, necrosis, necroptosis, pyroptosis, and ferroptosis (Fig. 5). These distinct modes of cell death represent critical late-stage pathological events that may influence the clinical trajectory of the disease.

#### Apoptosis

Apoptosis is a physiological, programmed form of cell death that plays a key role in maintaining tissue homeostasis. It is characterized by cell shrinkage, preservation of organelles, and chromatin condensation. During this process, acinar cells fragment into membrane-bound apoptotic bodies that are efficiently cleared by macrophages. Because cellular contents are not released, apoptosis does not elicit an inflammatory response.^250^ Three major apoptotic signaling pathways have been identified: the mitochondrial pathway, the death receptor pathway, and the ER pathway. In the mitochondrial pathway, apoptotic stimuli increase mitochondrial outer membrane permeability, leading to the release of cytochrome c into the cytoplasm. Cytochrome c then binds to apoptotic protease activating factor-1, activating caspase-9 and initiating the apoptotic cascade.^251^ The death receptor pathway is mediated by members of the TNFR superfamily, including Fas/FasL, TRAILR/TRAIL, and TNFR1/TNF. Ligand binding triggers the autocleavage of procaspase-8 into its active form, which initiates downstream caspase activation and the execution of apoptosis. ER stress can promote acinar cell apoptosis by activating the p53/AIFM2, PERK, and JNK pathways, thereby exacerbating the progression of AP.^252–254^ A study has shown that the apoptosis of pancreatic acinar cells occurs during SAP, peaking at 24 h before gradually decreasing.^255^ Intravenous injection of human placental mesenchymal stem cells effectively inhibits acinar apoptosis and thereby attenuates AP.^255^ However, some researchers suggest that acinar cell apoptosis may exert a protective effect during the course of AP.^256^ This apparent contradiction may reflect differences in the extent and context of acinar cell apoptosis. In MAP, apoptosis is predominant and may play a protective role, whereas in SAP, widespread necrosis is more prominent, with limited apoptosis observed.^257^ As apoptosis typically helps contain the inflammatory cascade, a shift from necrosis to apoptosis in SAP may represent a beneficial adaptation that reduces disease severity. This may help explain why enhancing apoptosis in certain contexts can attenuate the course of AP.

#### Necrosis

Cell necrosis is a passive form of cell death characterized by extensive membrane rupture and the release of DAMPs, which can trigger intense inflammatory responses and cause necrosis in adjacent tissues, thereby expanding necrotic regions and amplifying inflammation. A regulated form of necrosis, known as necroptosis, is a caspase-independent cell death mechanism that displays typical features of necrosis, including morphological alterations, subcellular changes, and metabolic dysfunction. In AP, necroptosis is mediated by receptor-interacting serine/threonine-protein kinases (RIPKs), particularly the RIPK1-RIPK3 axis and mixed lineage kinase domain-like protein (MLKL). In this pathway, RIPK3 phosphorylates MLKL, leading to its oligomerization and translocation to the plasma membrane to execute necroptosis.^251^ Necroptosis is considered a more aggressive form of cell death and may represent a predominant mode of acinar cell death in AP.^258^ Studies have shown that genetic deletion of RIPK3 or pharmacological inhibition of the RIPK1/RIPK3 pathway via necrostatin can significantly reduce AP severity. Notably, necrostatin remains effective even after the onset of pancreatitis, suggesting its therapeutic potential in limiting pancreatic injury.^258^ Thus, targeting necroptosis may offer a promising strategy for managing SAP. For example, AICAR, a direct activator of AMPK, has been reported to stabilize caspase-8 precursors, enhance RIPK3 degradation, and suppress necroptosis in pancreatic acinar cells, thereby attenuating disease severity and converting SAP in obese mice to a milder form.^259^ However, Boonchan et al.^260^ developed AP models using *RIPK3*^*−/−*^ or *MLKL*^*−/−*^ mice and reported that *RIPK3*- and *MLKL*-mediated necroptosis plays a protective role in AP, cautioning against the indiscriminate use of necroptosis inhibitors for treatment. These findings highlight the complexity of necroptosis in AP pathogenesis and underscore the need for further mechanistic studies.

#### Pyroptosis

Pyroptosis is a highly inflammatory form of programmed cell death that is regulated by pattern recognition receptors and executed via inflammasomes such as NLRP3. Caspase-1, as well as caspases -4, -5, and -11, cleave the substrate gasdermin D, releasing its N-terminal fragment. This fragment forms pores in the plasma membrane, leading to cell swelling, membrane rupture, and the release of proinflammatory cytoplasmic contents. Pyroptosis can proceed via the classical pathway, which is caspase-1 dependent, or the nonclassical pathway, which is mediated by caspases -4, -5, and -11.^251^ In hyperlipidemia-induced AP models, M1-polarized macrophages activate the classical caspase-1-dependent pathway through the secretion of cathepsin S, which contributes to inflammation and pancreatic injury. Inhibition of cathepsin S significantly alleviates symptoms in this model.^261^ In addition to cathepsin S, CTSB has also been implicated in promoting caspase-1-mediated pyroptosis by activating the NLRP3 inflammasome, further exacerbating pancreatic inflammation.^192^ These findings suggest that lysosomal pathways may play a critical role in regulating acinar cell death during AP, a possibility that warrants further investigation.

#### Ferroptosis

Ferroptosis is a recently identified form of regulated cell death, first proposed by Dr. Brent R. Stockwell at Columbia University in 2012.^251^ It is characterized by iron-dependent lipid peroxidation downstream of metabolic dysregulation. Mechanistically, ferroptosis involves the depletion of glutathione, a decrease in glutathione peroxidase 4 activity, and the consequent failure to eliminate lipid peroxides. As a result, Fe²⁺ catalyzes lipid oxidation, leading to the accumulation of reactive oxygen species (ROS) and triggering ferroptosis. Emerging evidence suggests that ferroptosis contributes to the pathogenesis of AP. The activation of nuclear factor erythroid 2–related factor 2 has been shown to upregulate glutathione peroxidase 4 and ferritin expression, thereby inhibiting ferroptosis in acinar cells and alleviating pancreatic injury in AP.^262^ Notably, the NADPH-dependent redox system is considered a central regulator of ferroptosis. In a model with acinar cell–specific deletion of isocitrate dehydrogenase 2, marked ferritin accumulation and pancreatic damage were observed. Conversely, coenzyme Q10 was found to mitigate ferroptosis and ferritin accumulation by activating the IDH2–NADPH pathway, thereby exerting a protective effect in AP.^263^

Importantly, the same type of death may have opposite biological effects at different stages and even in different severity of AP. For example, modest induction of acinar apoptosis in MAP may help control inflammation, whereas in SAP, cell necrosis should be prevented as much as possible. Necroptosis has been shown to have both proinflammatory and protective effects in different studies, suggesting that its regulatory mechanism is complex and space-time dependent. Similarly, ferroptosis is closely related to oxidative stress, and intervening in the Nrf2/GPX4 or NADPH metabolic pathways upstream is expected to be a new therapeutic strategy. In terms of pyroptosis, lysosomal enzymes (e.g., CTSB/CTSS) activate the NLRP3 inflammasome, which may form a key link with the inflammatory amplification ring between acinar cells and macrophages. ER stress plays an important role in the regulation of multiple modes of pancreatic acinar cell death in AP. For example, ER stress can promote acinar cell apoptosis by activating the p53/AIFM2 axis and the PERK–JNK pathway,^252–254,264^ induce caspase-1-dependent pyroptosis via activation of the PERK pathway,^265^ and trigger necroptosis in pancreatic acinar cells by enhancing CTSB maturation and activating the PKCα–JNK–c-Jun cascade.^266^ On the basis of the above mechanism, targeting ER stress–related pathways to modulate acinar cell death may represent a promising therapeutic strategy for improving the pathological progression of AP.

### Inflammatory responses

**Fig. 6:**
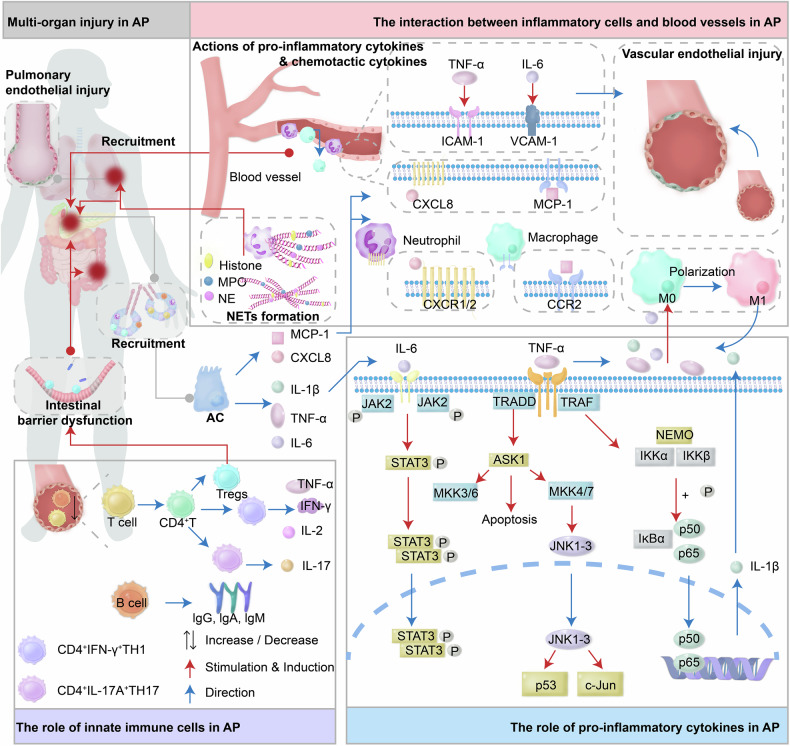
Cascade of inflammatory responses in acute pancreatitis. In the early phase of AP, injured acinar cells increase microvascular permeability by releasing proinflammatory cytokines (e.g., TNF-α, IL-6, and IL-1β) and upregulating adhesion molecules such as ICAM-1 and VCAM-1 on vascular endothelial cells. Moreover, they secrete chemokines (e.g., CXCL8 and MCP-1) that recruit neutrophils and monocytes into the pancreatic tissue. Within the pancreas, TNF-α, IL-6, and IL-1β not only activate neutrophils to release effector molecules such as MPO and histones, leading to the formation of NETs but also drive resident macrophages toward a proinflammatory phenotype via key signaling pathways such as the JAK/STAT and NF-κB pathways. These activated macrophages further amplify local inflammation by producing abundant inflammatory mediators. Simultaneously, these cytokines and immune cells may enter systemic circulation, potentially contributing to acute lung injury. In parallel, peripheral T and B lymphocytes migrate to the pancreas and undergo functional differentiation within the AP-associated microenvironment. CD4⁺ T cells differentiate into Th1 and Th17 subsets, releasing cytokines such as IFN-γ, IL-2, TNF-α, and IL-17, which together intensify the inflammatory response. Additionally, circulating Tregs may migrate to the gut, where they disrupt intestinal barrier integrity through multiple mechanisms, worsening mucosal injury and potentially promoting bacterial translocation and the onset of systemic inflammatory response syndrome. AP acute pancreatitis, CCR2 C-C chemokine receptor type 2, CD4⁺ cluster of differentiation 4 positive, CXCL8 C-X-C motif chemokine ligand 8, CXCR1/2 C-X-C motif chemokine receptor 1/2, ICAM-1 intercellular adhesion molecule 1, IFN-γ interferon gamma, IgA/IgG/IgM immunoglobulin A/G/M, IKKα/IKKβ inhibitor of nuclear factor kappa-B kinase subunit alpha/beta, IL-1β/IL-2/IL-6/IL-17 interleukin-1 β/2/6/17, JAK2 janus kinase 2, M1 M1-polarized macrophage, MCP-1 monocyte chemoattractant protein-1, MKK3/6 MAPK kinase 3/6, MKK4/7 MAPK kinase 4/7, MPO myeloperoxidase, NE neutrophil elastase, NETs neutrophil extracellular traps, NF-κB nuclear factor kappa-light-chain-enhancer of activated B cells, NEMO NF-κB essential modulator, p50 nuclear factor NF-κB p50 subunit, p53 tumor protein p53, p65 nuclear factor NF-κB p65 subunit, STAT3 signal transducer and activator of transcription 3, T cell T lymphocyte, TH1 T helper 1 cell, TH17 T helper 17 cell, TNF-α tumor necrosis factor alpha, TRADD TNFR1-associated death domain protein, TRAF2 TNF receptor-associated factor 2, Tregs regulatory T cells, VCAM-1 vascular cell adhesion molecule 1

During the early injury phase of AP, pancreatic parenchymal cells release DAMPs; proinflammatory cytokines such as IL-6, tumor necrosis factor α (TNF-α), and interleukin-1β (IL-1β); and chemokines, including IL-8 and monocyte chemoattractant protein-1 (MCP-1).^267,268^ As AP progresses, inflammatory mediators further amplify both local and systemic inflammatory responses by activating signaling pathways such as the NF-κB pathway and recruiting immune cells, including neutrophils and macrophages, which are critical drivers of disease prognosis (Fig. 6). Importantly, this stage of hyperinflammation is frequently accompanied by compensatory anti-inflammatory response syndrome, which contributes to an immunosuppressive state and may promote the development of secondary pancreatic necrosis. Therefore, a deeper understanding of the regulatory mechanisms underlying inflammation in AP and the identification of key targets within the inflammatory cascade may help optimize the pancreatic microenvironment and improve patient outcomes.

#### Role of proinflammatory cytokines

IL-6 is a key cytokine involved in the pathogenesis of AP. As a member of the gp130 ligand family, IL-6 transduces signals by binding to its membrane-bound receptor and the ubiquitously expressed gp130 receptor.^269^ IL-6 signals through two distinct pathways: classical and trans-signaling. In the classical pathway, IL-6 binds to gp130, a 130-kDa signal transducer that is expressed primarily on hepatocytes and selected leukocyte subsets.^267^ In the trans-signaling pathway, which increases the bioavailability of circulating IL-6, IL-6 forms a complex with its soluble receptor. Both pathways ultimately activate the Janus kinase/signal transducer and activator of transcription (JAK/STAT) pathway,^270^ which is critical for mounting immune responses, controlling infection, and maintaining immune homeostasis.^267^ Clinical studies have shown that IL-6 levels typically peak within 24–48 h following the onset of AP. Notably, IL-6 levels are significantly higher in patients with SAP than in those with MAP, supporting its potential role as a biomarker for early prediction and severity discrimination in AP.^271,272^ In addition, elevated serum IL-6 levels are closely associated with early complications of AP, including acute necrotizing effusion, acute peripancreatic effusion, pleural effusion, and ascites.^273^ Further investigations have demonstrated that IL-6 contributes to neutrophil infiltration by upregulating the expression of adhesion molecules such as ICAM-1 and VCAM-1^274^ and promotes the polarization of proinflammatory M1 macrophages,^275^ both of which are implicated in systemic inflammation and distant organ injury. Given the central pathogenic role of IL-6 in AP, both clinical and experimental studies suggest that targeting the IL-6 signaling pathway—particularly its trans-signaling arm—may represent a promising therapeutic approach. Tocilizumab, an IL-6 receptor antagonist widely used to treat rheumatoid arthritis and other inflammatory diseases, could theoretically attenuate the proinflammatory effects of IL-6 in AP. However, its safety and efficacy in this context have not yet been evaluated in clinical trials.^276^ Importantly, systemic blockade of IL-6 may also impair tissue repair and disrupt immune homeostasis maintained by classical IL-6 signaling, thereby potentially increasing the risk of infection.

TNF-α, a proinflammatory cytokine, promotes immune cell recruitment and cell death by inducing the expression of cytokines, chemokines, adhesion molecules, cyclooxygenase-2, and inducible nitric oxide synthase or by activating signaling pathways such as the NF-κB, STAT, and PPARs pathways.^277^ Its biological effects are mediated by two distinct surface receptors: TNFR1 (p55) and TNFR2 (p75). TNF-α, a transmembrane protein, preferentially activates TNFR2, which is generally associated with cell survival and immune regulation.^278^ In contrast, when cleaved from the membrane, soluble TNF-α activates TNFR1, triggering apoptosis or the NF-κB/MAPK pathway, thereby promoting inflammation and tissue edema.^278^ A clinical study revealed elevated serum levels of TNF-α and its soluble receptors in patients with AP,^279^ although the levels of TNF-α or soluble TNF-α alone have limited predictive value for early septic shock in SAP patients.^280^ In the early stages of AP, TNF-α released from injured pancreatic acinar cells^277^ exacerbates oxidative stress by converting xanthine dehydrogenase to xanthine oxidase, increases the permeability of capillary endothelial and postcapillary venule cells, and upregulates the expression of adhesion molecules such as selectin and ICAM-1, thereby facilitating leukocyte rolling, adhesion, and infiltration into pancreatic tissue.^281^ Additionally, pancreatic enzymes such as carboxypeptidase A and lipase can activate resident macrophages, further inducing TNF-α production.^282^ The resulting amplification loop, involving neutrophil- and macrophage-derived TNF-α, intensifies the local inflammatory response in the pancreas. Thus, simultaneous inhibition of TNF-α production and xanthine oxidase activity markedly reduces both local and systemic inflammatory responses in AP and is associated with decreased mortality.^283^ For example, XPro1595, a newly developed biologic that selectively inhibits soluble TNF-α, has been shown to significantly improve pancreatic pathology in a mouse model of caerulein-induced AP.^278^ In addition to its local effects, TNF-α released from the pancreas contributes to inflammation in distant organs such as the lungs, promoting events such as endothelial activation, leukocyte recruitment, and capillary leakage. It is therefore considered a key mediator in the pathogenesis of AP associated acute lung injury (AP-ALI). For example, TNF-α modulates lung function in AP by interacting with aquaporin-1, and hypoxia has been shown to alleviate AP-ALI by increasing aquaporin-1 expression and suppressing TNF-α levels.^284^ Similarly, thalidomide attenuates AP-ALI in rats by downregulating TNF-α expression through the inhibition of the NF-κB signaling pathway.^285^ In clinical research, infliximab, an anti-TNF-α monoclonal antibody, has been shown to significantly reduce the severity and mortality of pancreatitis in animal models. The ongoing RAPID I clinical trial is currently evaluating its safety and efficacy in patients with AP.^286^ However, high-quality clinical evidence in human populations remains limited. Like IL-6 inhibitors, the systemic immunosuppressive nature of infliximab may increase susceptibility to infections. Moreover, rare cases of pancreatitis associated with TNF-α inhibitors underscore the need for careful evaluation of potential adverse events.

Aseptic necrosis of acinar cells is a key event in AP, and IL-1β is a central effector cytokine involved in sterile injury mediated by the innate immune response, which is typically produced by monocytes and macrophages.^287^ The precursor form of IL-1β is induced by DAMPs via Toll-like receptor 9 and is subsequently cleaved and activated by caspase-1.^288^ Recent studies have shown that IL-1β mediates pancreatic inflammation through multiple mechanisms, including promoting neutrophil infiltration into inflamed tissue and stimulating the production of other proinflammatory cytokines and chemokines,^289^ which are essential for the progression of pancreatic and remote organ injury in AP.^290^ Mechanistically, IL-1β amplifies the inflammatory cascade by activating MAPKs and NF-κB, thereby promoting further cytokine and chemokine release.^291^ Elevated serum IL-1β levels are associated with pleural effusion, an early complication of AP,^273^ although other studies have reported no significant correlation between IL-1β gene polymorphisms and AP susceptibility.^292^ To date, no clinical studies have reported the use of IL-1β inhibitors for the treatment of AP.

In summary, IL-6, TNF-α, and IL-1β are recognized as key cytokines involved in the pathogenesis of AP, and suppressing their expression can significantly improve the inflammatory microenvironment. However, current strategies for targeted intervention remain limited. Moving forward, it may be more effective to focus on the dynamic regulatory networks of inflammatory mediators to increase the therapeutic efficacy of clinical interventions.

#### Role of chemokines

In recent years, chemokines—small (8–10 kDa), soluble proteins—have been increasingly recognized for their role in the pathogenesis of AP, particularly through mediating inflammatory cell adhesion, integrin activation, and leukocyte transendothelial migration.^293^ Chemokines are generally classified into two major subfamilies: C-X-C and C-C. C-X-C chemokines, such as CXCL8, primarily target neutrophils, whereas C-C chemokines, such as MCP-1, predominantly recruit monocytes.^294^ In the following section, we briefly outline the roles of these two representative chemokines in the context of AP.

CXCL8 (also known as IL-8), a prototypical C-X-C chemokine, is produced by monocytes, macrophages, neutrophils, natural killer cells, and chemotactically active adipocytes.^295,296^ It has also been identified as a secretory product of acinar cells, pancreatic ductal cells, and activated human periacinar myofibroblasts.^295^ Notably, CXCL8 is among the earliest detectable chemokines in the serum of patients with AP, where it acts as both a chemoattractant and an activator of neutrophils.^297^ Its effects are primarily mediated through two neutrophil receptors, CXCR1 and CXCR2.^298^ During the early phase of AP, injured acinar cells secrete CXCL8, which contributes to neutrophil activation and recruitment, thereby promoting the downstream inflammatory cascade.^299^ Several studies have reported that individuals carrying the IL-8-251T/A polymorphism may have increased susceptibility to AP.^300,301^ However, a population-based study conducted in Suzhou did not find a significant association between IL-8‒251 polymorphisms and AP risk,^302^ possibly due to limitations such as a small sample size and a narrow population range. Moreover, elevated serum IL-8 levels have been linked to complications of SAP, including renal and respiratory failure.^303^ Moreover, IL-8 plays an important role in SAP complicated by abdominal compartment syndrome. Early initiation of continuous veno-venous hemofiltration has been shown to significantly reduce serum IL-8 levels and lower intra-abdominal pressure in patients with SAP and abdominal compartment syndrome.^304^ IL-8 is a potent proinflammatory cytokine, similar to IL-6, and the combination of IL-6 and IL-8 has good predictive value for SAP.^305^ Both cytokines can drive the progression from localized pancreatic inflammation to a systemic inflammatory response, and elevated levels of IL-6 and IL-8 are associated with an increased risk of SAP complicated by ALI.^306^ Therefore, strategies aimed at suppressing IL-8 release may help reduce the severity and mortality of SAP. For example, glutamine has been reported to alleviate SAP by downregulating IL-8-mediated proinflammatory cytokine production.^307^ Currently, targeted CXCR1/2 antagonists, such as Reparixin, have not been evaluated in clinical studies of AP, although they have shown promising anti-inflammatory effects in transplant-related settings.^308^ Similarly, HuMax-IL8 (BMS-986253), an anti-CXCL8 antibody, has entered phase I trials for advanced solid tumors and chronic inflammatory diseases; however, no AP-specific data are currently available.^309^ Notably, IL-8 functions as an acute-phase reactant, and its transient overexpression may serve as a protective response. Therefore, it is essential to carefully define the appropriate patient population and timing of intervention in future studies.

Unlike IL-8, MCP-1 is a member of the C-C chemokine family and is induced by proinflammatory stimuli such as TNF-α and IFN-γ and by lipopolysaccharides in a variety of cell types, including fibroblasts, endothelial cells, smooth muscle cells, keratinocytes, hepatocytes, monocytes/macrophages, and lymphocytes.^310^ MCP-1 contributes to the recruitment of monocytes and macrophages and to the activation of lymphocytes during inflammation.^311^ Rat pancreatic acinar cells have been shown to secrete MCP-1 independently of stimulation by circulating leukocytes, and certain peptides—such as caerulein—can directly induce MCP-1 expression in acinar cells.^312,313^ In a rat model of AP, plasma MCP-1 levels were significantly elevated as early as 6 h following pancreatic duct injection of sodium taurocholate,^314^ suggesting that acinar-derived MCP-1 acts as an early inflammatory mediator in AP. A clinical study demonstrated that elevated serum MCP-1 levels at admission are strongly associated with the risk of developing SAP, and urinary MCP-1 may also serve as a potential predictive biomarker.^315^ Rau et al.^316^ reported significantly higher serum MCP-1 levels in patients with local complications and/or distant organ failure. However, a population-based study in Suzhou suggested that the MCP-1-2518 AA genotype may be protective against AP, whereas individuals with a higher frequency of the G allele were more susceptible to disease onset.^302^

Therefore, inhibiting MCP-1 activity may alleviate the severity and complications of SAP. For example, a plasmid expression vector carrying a dominant-negative mutant of the MCP-1 gene has been shown to significantly reduce the incidence of severe pancreatitis and improve the 48-h survival rate following disease onset.^310^ Additionally, bindarit—a compound that preferentially suppresses MCP-1 production both in vitro and in vivo—has demonstrated prophylactic and therapeutic efficacy in SAP mouse models, significantly lowering pancreatic MCP-1 levels and providing protection against AP.^313,317^ Moreover, MCP-1, a key chemokine in central nervous system inflammation, can disrupt blood‒brain barrier integrity by targeting tight junctions. Notably, nifedipine, an MCP-1 antagonist, has been reported to improve blood‒brain barrier function in SAP rats by downregulating MCP-1 expression.^311^ In clinical studies, monoclonal antibodies targeting MCP-1, such as Carlumab, have been investigated in trials for tumors and idiopathic pulmonary fibrosis; however, their efficacy remains uncertain.^318,319^ Moreover, MCP-1 is a representative redundant chemokine that may be partially compensated for by other CC chemokines, potentially limiting its therapeutic consistency. In clinical practice, adjustments to MCP-1-targeted strategies—such as accounting for the monocyte-to-neutrophil ratio, oxygenation index, and concurrent medications—should be made with careful consideration of each patient’s comorbidities.

#### Role of innate immune cells

Immune cell infiltration into the pancreas is a critical event in the development of AP. As previously described, damaged acinar cells secrete MCP-1, which recruits CCR2-positive circulating inflammatory monocytes/macrophages into the pancreas. These recruited monocyte/macrophage populations, together with tissue-resident macrophages, amplify the local inflammatory response by releasing additional inflammatory mediators, cytokines, and growth factors into the pancreatic microenvironment.^320,321^

Macrophages are the most abundant immune cells in the pancreas during the early stages of inflammation in AP. Most macrophages, including tissue-resident cells and bone marrow–derived monocytes, can polarize into either proinflammatory M1 or anti-inflammatory M2 phenotypes, depending on the nature of the stimulus and changes in the local microenvironment.^275,322^ M1 macrophages promote immune activation and contribute to tissue injury by producing nitric oxide, ROS, various chemokines (CXCL2, CXCL4, and CCL5), and proinflammatory cytokines such as TNF-α, IL-1β, and IL-6. In contrast, M2 macrophages are associated with tissue repair and fibrosis through the secretion of anti-inflammatory mediators, including IL-10, arginase-1, CCL24, and CCL22.^323^ In the early stages of AP, necrotic acinar cells create a unique metabolic microenvironment that promotes M1 polarization of macrophages.^324^ This process is mediated by the release of various factors, including DAMPs^325^ and chemokines such as CXCL10.^326^ These signals influence resident macrophages located near acinar cells,^297^ thereby exacerbating the progression of pancreatitis. Additionally, during AP, acinar cells regulate the MARCH3/NLRP3 axis via high expression of miR-24-3p, which promotes M1 polarization and pyroptosis of peritoneal macrophages, contributing to abdominal inflammation.^327^ The proton pump inhibitor esomeprazole has been shown to attenuate pancreatic tissue injury by suppressing macrophage activation and lowering the serum levels of inflammatory cytokines such as IL-1β and TNF-α.^328^ In addition, the antifibrotic drug pirfenidone has shown good efficacy in a variety of AP animal models and can reduce the release of proinflammatory factors by acinar cells and macrophages and increase the secretion of the anti-inflammatory factor IL-10 by macrophages. Its rigorous preclinical research design provides an important reference for translational medicine.^329,330^ Furthermore, polarized M1 alveolar macrophages have been implicated in pancreatitis-associated lung injury.^331^ Therefore, targeting macrophage M1 polarization or suppressing the release of inflammatory mediators—such as semapimod (CNI-1493), which inhibits the production of nitric oxide and proinflammatory cytokines in macrophages—is promising for improving the inflammatory microenvironment in AP. Semapimod has shown efficacy in reducing inflammation and mortality in early animal studies,^332,333^ although its clinical benefits remain uncertain.^334^ Additionally, ulinastatin, which downregulates macrophage-driven inflammatory pathways (e.g., NF‑κB and MMPs), has been reported to attenuate pancreatic tissue injury.^335,336^ In the early phase of AP, macrophages predominantly adopt the M1 phenotype, releasing proinflammatory cytokines such as TNF‑α and IL‑1β that exacerbate tissue damage. As the disease progresses, macrophages can shift toward the M2 phenotype, contributing to tissue repair and fibrosis. Thus, careful modulation of macrophage polarization across different disease stages may offer a promising strategy to coordinate inflammatory resolution and tissue recovery in AP.

Neutrophils, which constitute 60–70% of circulating leukocytes,^337^ are key inflammatory cells involved in the progression of AP. They are among the first immune cells recruited to the site of pancreatic injury.^338^ Within three hours of an initial pancreatic insult, damaged acinar cells recruit neutrophils by releasing chemokines such as CXCL8 and CXCL1.^297^ Upon activation, neutrophils release DNA-based reticular structures composed of histones, HMGB1, cathepsin G, and neutrophil elastase, collectively known as neutrophil extracellular traps (NETs).^339^ These NETs help trap and eliminate pathogens, contributing to the rapid control of infection in vivo.^340^ However, accumulating evidence suggests that excessive and dysregulated NET formation may worsen pancreatic injury by activating trypsinogen within acinar cells.^341^ In addition, many neutrophils can migrate to the lungs via the bloodstream in response to chemokine signaling, where the release of NETs and matrix metalloproteinases can disrupt alveolar capillaries, contributing to ALI, a hallmark of AP-ALI.^325,342^ Notably, treatment with DNase I in SAP mouse models has been shown to reduce neutrophil infiltration in both the pancreas and lungs, significantly alleviating tissue damage.^343^ Intervention strategies targeting these mechanisms—such as DNase I, which degrades NETs—have significantly reduced pancreatic and pulmonary injury in animal models of AP.^343,344^ Similarly, the neutrophil elastase inhibitor Sivelestat has been shown to improve the oxygenation index in acute respiratory distress syndrome patients, shorten the duration of mechanical ventilation, and reduce the length of stay in the ICU.^345^ Although these agents have not yet been widely adopted in clinical practice for AP, they provide a promising foundation for therapeutic intervention in AP-ALI.

#### Role of adaptive immune cells

In addition to the involvement of innate immune cells, aberrant activation of adaptive immune cells also plays a key role in the pathogenesis of AP. Adaptive immune cells are broadly categorized into two subtypes on the basis of their surface markers and functional characteristics: T lymphocytes (T cells) and B lymphocytes (B cells). B cells, which are derived from pluripotent stem cells in the bone marrow, differentiate into plasma cells upon antigen stimulation and are responsible for humoral immunity through the secretion of immunoglobulins such as IgG, IgA, and IgM. A study has shown that peripheral B-cell levels are lower in patients with AP than in healthy controls.^346^ Moreover, B-cell frequency has been associated with disease severity, with patients suffering from MSAP to SAP exhibiting higher percentages of total B cells than those with MAP.^347^ Measuring B-cell levels may thus provide a useful tool for the early assessment of AP severity. Additionally, peripheral B cells are closely related to disease progression, as their frequency positively correlates with CRP levels and length of hospital stay, suggesting that these cells are potential markers for evaluating therapeutic response.^348^ T cells, primarily CD4⁺ T cells, originate in the thymus and are essential for maintaining systemic immune regulation. An analysis of pancreatic tissues from patients who died of AP and those with non-pancreatic acute abdominal conditions revealed a marked reduction in T and B-cell expression in perinecrotic pancreatic tissue, which may indicate a poorer prognosis in AP patients.^349^ Furthermore, early changes in CD4⁺ T and CD19⁺ B-cell levels may help predict the risk of organ failure. In addition, early changes in CD4⁺ T cells may serve as a predictive marker for the risk of organ dysfunction. Studies have shown that peripheral blood CD4⁺ T-cell levels are significantly lower in patients with AP who develop organ dysfunction than in those without such complications.^350^

In the early stages of AP, the number of circulating T cells, including both the CD4⁺ and CD8⁺ subsets, is markedly reduced.^351^ A decrease in peripheral CD4⁺ T cells has been associated with the development of abdominal compartment syndrome in patients with SAP, suggesting its potential as a predictor of abdominal compartment syndrome.^350^ CD4⁺ T cells differentiate into various effector subtypes, including Th1, Th2, Th9, Th17, regulatory T cells (Tregs), and T follicular helper cells.^352^ Although AP significantly impairs the activity of peripheral CD4⁺ T cells, their recruitment into pancreatic tissues has been reported.^353^ These CD4⁺ T cells can exacerbate inflammation and tissue injury by differentiating into CD4⁺/IFN-γ⁺ Th1 and CD4⁺/IL-17A⁺ Th17 cells.^354^ Th1 cells contribute to cellular immune responses by producing IFN-γ, TNF-α, and IL-2, whereas Th17 cells amplify inflammatory cascades and pancreatic injury through the secretion of IL-17 and the modulation of chemokine and inflammatory molecule expression.^355^ Thus, the decline in the number of circulating T cells during AP may be partly explained by their migration into inflamed pancreatic tissue. In addition, T-cell depletion has been linked to increased apoptosis mediated by the Fas/FasL signaling pathway, as significantly elevated expression of Fas and FasL has been detected in splenic lymphocytes from SAP mice.^350,356^ Tregs have also been implicated in the pathogenesis of infectious pancreatic necrosis by impairing the duodenal barrier and facilitating bacterial translocation to necrotic pancreatic tissue. Therefore, the modulation of Tregs may represent a potential therapeutic strategy to mitigate infection and necrosis in SAP. For example, butyrate has been shown to attenuate intestinal injury in SAP by promoting the differentiation of Tregs.^350^ An analysis of peripheral blood samples from AP patients with different clinical outcomes revealed that a significantly greater percentage of circulating Tregs was observed in patients with poorer prognoses, potentially due to Treg-mediated suppression of systemic immune responses.^357^ However, prophylactic depletion of Tregs via intraperitoneal diphtheria toxin injection was shown to stabilize the intestinal immune barrier and reduce bacterial translocation to the inflamed pancreas but paradoxically intensified the proinflammatory response in experimental AP models.^358^ These findings suggest that complete depletion of Tregs may not be an optimal strategy for managing AP. Additionally, a reduction in CD4⁺CD25⁺ Treg levels can increase the activity of effector CD4⁺ T cells and exacerbate local inflammation.^359^ In contrast, increasing the proportion of CD4⁺CD25⁺ Tregs has been shown to suppress T-cell proliferation and modulate immune responses. For example, nicotine has been reported to alleviate severe experimental AP by enhancing the immunoregulatory function of CD4⁺CD25⁺ Tregs.^360^ In summary, T cells not only contribute to the amplification of early tissue injury but also influence immune status and the risk of secondary infections in the later stages of AP. Currently, no targeted therapies for T cells are available in clinical practice. Therefore, future research should aim to elucidate strategies for the precise regulation of T-cell recruitment, activation, subset balance, and immune function.

In summary, during the early pathological stages of AP, abnormal zymogen activation, calcium overload, and impaired autophagy constitute three core mechanisms driving disease progression. Variations in acinar cell death modes directly influence the clinical severity of AP. Notably, the inflammatory response not only contributes to early local pancreatic inflammation but also plays a pivotal role in the subsequent development of MODS. Although basic research has shown that targeting these pathological processes can help alleviate clinical manifestations—such as reducing pancreatic edema, attenuating inflammation, and limiting organ dysfunction—most of these strategies remain limited to cellular or animal models and have not yet undergone large-scale clinical validation. Given the non-regenerative nature of acinar cells and the progressive nature of necrosis once initiated, identifying effective therapeutic strategies to delay or prevent acinar cell necrosis holds significant clinical relevance for halting disease deterioration and improving prognosis. Future research should therefore focus on how alterations in the acinar microenvironment—such as osmotic pressure, pH, and electrolyte homeostasis—influence cellular function. Special attention should also be given to the stability of acinar secretory vesicles and membrane fusion dynamics. Elucidating their roles in the abnormal activation of zymogens may help uncover the initiating pathological events in AP, thereby enabling the disruption of downstream inflammatory cascades.

## Therapeutic strategies

**Fig. 7:**
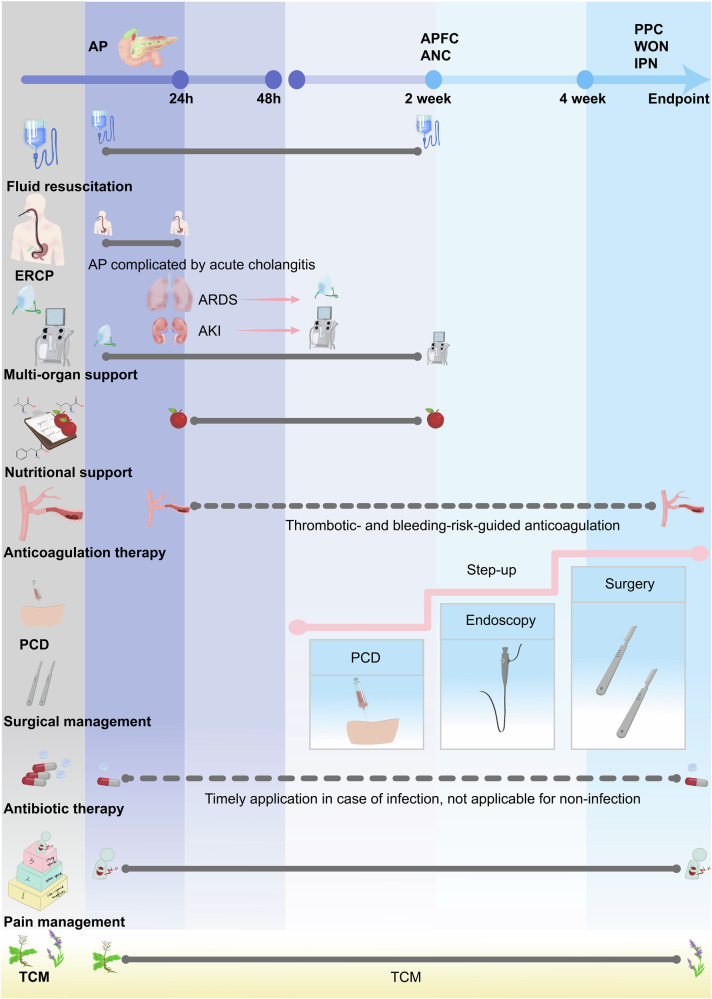
Selection of intervention and treatment timing in acute pancreatitis patients. This figure illustrates the timing and sequence of therapeutic interventions during the clinical course of acute pancreatitis. The treatment strategies include both conventional Western medical approaches and adjunctive modalities derived from TCM. The application of each intervention is clearly mapped to specific stages of disease progression. AKI acute kidney injury, ANC acute necrotic collection, AP acute pancreatitis, APFC acute peripancreatic fluid collection, ARDS acute respiratory distress syndrome, ERCP endoscopic retrograde cholangiopancreatography, IPN infected pancreatic necrosis, PCD percutaneous catheter drainage, PPC pancreatic pseudocyst, TCM traditional Chinese medicine, WON walled-off necrosis

The course of AP can be divided into early and late stages, which overlap with each other, corresponding to the two death peaks in the course of AP. The early stage is characterized by SIRS and organ dysfunction, and although local complications may occur at this stage, they are not major determinants of disease severity. The later stage is characterized by persistent SIRS, organ dysfunction, and serious complications. In terms of treatment, the strategy includes fluid resuscitation, pain management, nutritional support, and symptomatic treatment for the cause and early complications. In the later stage of the disease course, serious local complications, including pancreatic pseudocyst, IPN, walled-off necrosis (WON), bleeding, gastrointestinal fistula, etc., are important determinants of the severity of the disease (Fig. 7 and Table 3).

**Table 3:** Comparative analysis of the main treatment measures for acute pancreatitis

Phrase	Intervention measures	Timing of intervention	Adaptation stage	Precautions	Drawbacks
Early treatment	Fluid resuscitation	Initiate intervention as early as possible after AP diagnosis^90,176,361^	Reduced circulating blood volume	Hemodynamic status should be reassessed regularly to avoid fluid overload^176,361^	Overreliance on laboratory parameters such as hematocrit
	Nutritional support	Intervention within 24–72 h after disease onset^366,367,383^	MSAP and SAP^377^	Enhanced monitoring is required in patients with malabsorption symptoms	Careful selection of enteral nutrition formulas (elemental, semi-elemental, or polymeric)
	Pain management	Initiate intervention upon onset of pain^388^	Suitable for all AP patients with pain	Analgesic strategies are graded according to the degree of pain^389^	May prolong hospital stay; long-term effects remain to be clarified^390^
	Antibiotic therapy	Timely application in case of infection, not applicable for non-infection^10,92,125,366^	Confirmed or highly suspected infection^401^	Antibiotic duration should be limited to 7–14 days^402^; adjust the treatment plan timely based on the results of the bacterial culture^92,401^	Prophylactic antibiotics are not recommended unless infection is clearly documented
Late treatment	PCD	Necrotic tissue liquefies and forms a well-defined encapsulation (onset ≥4 weeks) ^429^	Infected pancreatic and/or peripancreatic necrosis	A retroperitoneal approach is preferred to minimize the risk of intraperitoneal infection spread^431^	Should be performed under ultrasound or CT guidance by an experienced interventional team
	Endoscopy	ERCP (within 24 h after the diagnosis of gallstone pancreatitis complicated with acute cholangitis); Endoscopic drainage (mature wall of pancreatic necrosis, onset >4 weeks)^434^	WON or pancreatic pseudocyst adjacent to the stomach or duodenum^432,433^	Once gallstones are removed, ERCP should be terminated immediately; access should be established only when the necrotic cavity is adjacent to the gastrointestinal wall without intervening vital structures	Advanced equipment and professional operators are needed; inability to resolve abscesses far from the gastrointestinal tract
	Minimally invasive surgery	Recommended after 4 weeks, when necrosis is enveloped in mature granulation tissue, unless life-threatening complications (e.g., ACS) occur	Primary wall necrosis; retrogastric necrosis extending along the left paracolic groove; entral pancreatic necrosis^442^	Risk of intra-abdominal infection	Limited surgical area^401^; repeated interventions are usually required
	Open surgery		Extensive necrosis with multilocular abscess; progressive organ failure due to ACS; euptured large pseudoaneurysm	Surgery during the peak systemic inflammatory phase of pancreatitis may exacerbate inflammation and trigger organ dysfunction	Highly invasive;sometimes reoperation or ongoing lavage is needed, affecting the patient’s long-term quality of life^446^

*ACS* abdominal compartment syndrome, *AP* acute pancreatitis, *MSAP* moderately severe acute pancreatitis, *PCD* percutaneous catheter drainage, *SAP* severe acute pancreatitis, *WON* walled-off necrosis

### Early treatment of acute pancreatitis

#### Fluid resuscitation

Patients with AP often experience fluid deficits due to intravascular volume depletion, which results from pancreatic, peripancreatic, and systemic edema; vomiting; reduced oral intake; and local inflammation. This hypovolemic state can lead to tissue hypoperfusion, thereby increasing the risk of morbidity and mortality.^90,176,361^ Early fluid resuscitation has been shown to significantly improve perfusion. Clinical guidelines from multiple authoritative organizations recommend initiating fluid resuscitation as soon as AP is diagnosed. The intravascular volume status should be assessed through monitoring vital signs, urine output, and BUN and hematocrit levels.^176,361^ Additionally, repeated evaluations of hemodynamic status during treatment are essential to guide fluid management and to detect potential complications such as tissue edema or organ dysfunction due to fluid overload.

The primary fluid resuscitation options for AP include crystalloid and colloid solutions, with lactated Ringer’s solution and normal saline being the most commonly used. Several studies have demonstrated that lactated Ringers’ solution offers significant advantages over normal saline, such as reduced disease severity, lower rates of ICU admission, and shorter hospital stays. These benefits may be attributed to its electrolyte composition being more physiologically similar to that of plasma and its potential to mitigate SIRS.^175,362^ For example, a meta-analysis of three randomized controlled trials reported that using lactated Ringer’s solution during the initial resuscitation phase was associated with a lower risk of SIRS.^363,364^ Although colloid solutions—such as hydroxyethyl starch—theoretically expand intravascular volume via oncotic pressure and may improve hypoalbuminemia, clinical data do not support a benefit in patients with AP or sepsis in the ICU. Consequently, the American Gastroenterological Association (AGA) explicitly advised against the use of hydroxyethyl starch.^365,366^ Similarly, the American College of Gastroenterology recommends lactated Ringer’s solution on the basis of current evidence, emphasizing its potential anti-inflammatory effects.^92,363,364,367^ However, excessive fluid administration—regardless of fluid type—may increase the risk of complications such as abdominal compartment syndrome, sepsis, and the need for mechanical ventilation.^368,369^ The ongoing WATERLAND trial, anticipated to conclude in 2026, is expected to provide higher-quality evidence by comparing the clinical outcomes of different crystalloid fluids.^370^

In the fluid management of AP, balancing the rate of fluid administration with the risk of complications remains a long-standing challenge. The central concern is how to optimize tissue perfusion while minimizing the risk of iatrogenic fluid overload.^371^ Recent evidence from the WATERFALL trial—a multicenter randomized study involving 249 patients—offers important insights. The participants were randomized into an aggressive resuscitation group (20 mL/kg rapid bolus followed by 3 mL/kg/h maintenance) and a moderate resuscitation group (hypovolemic patients received a 10 mL/kg bolus followed by 1.5 mL/kg/h maintenance). Interim analysis revealed no significant difference in the incidence of moderate to severe pancreatitis between the two groups. However, the aggressive resuscitation group had a significantly greater risk of fluid overload and prolonged median hospital stays, leading to early termination of the trial due to safety concerns.^371,372^ These findings are consistent with earlier studies indicating that uncontrolled large-volume fluid resuscitation markedly increases the risk of mechanical ventilation, abdominal compartment syndrome, and mortality.^368^ Furthermore, forced hemodilution—targeting a hematocrit of <35% within 48 h—was associated with a twofold increase in the incidence and 28-day mortality of sepsis.^373^ A recent meta-analysis further supports these concerns, showing that aggressive resuscitation strategies are significantly associated with higher rates of organ failure, ALI, and acute kidney injury. The proposed mechanisms include exacerbated capillary leakage, increased dissemination of inflammatory mediators, and increased abdominal pressure, leading to hypertension.^374,375^ Additionally, aggressive fluid resuscitation in patients with preexisting renal insufficiency or heart failure may increase the risk of volume overload, which can present as pulmonary edema, hypoxia, or intra-abdominal hypertension.^373^ In light of this evidence, current guidelines recommend an individualized, goal-directed, and moderate resuscitation approach: for hypovolemic patients, a rapid infusion of 10 mL/kg is initiated, followed by maintenance fluids at 1.5 mL/kg/h. The regimen should be reassessed at least every 6 h and adjusted on the basis of clinical parameters (heart rate <120 beats/min, mean arterial pressure 65–85 mmHg, and urine output >0.5 mL/kg/h), biochemical markers (hematocrit <44%), and imaging findings.^371,373,376^ Furthermore, emerging data caution against overreliance on laboratory values such as hematocrit or rigid implementation of fixed infusion protocols. Instead, real-time assessment of perfusion status using parameters such as heart rate, mean arterial pressure, and urine output should guide fluid management to achieve a balance between adequate tissue oxygenation and the risk of fluid overload.^175,372,373^

The above section provides a systematic review of the current research progress on crystalloid selection and infusion rates in fluid resuscitation for AP, underscoring the importance of individualized and goal-directed treatment. Nevertheless, several unresolved issues persist in clinical practice. First, although lactated Ringer’s solution has demonstrated superior anti-inflammatory effects and better electrolyte stability than normal saline has in multiple studies, its safety profile has not been fully evaluated in specific patient populations, such as those with hepatic insufficiency or lactate metabolism disorders. In such cases, the fluid type should be selected with particular caution. Second, while trials such as the WATERFALL study emphasize the importance of avoiding over-resuscitation, the lower threshold for “moderate resuscitation” remains poorly defined. Currently, there is no clear consensus on whether patients with mild to moderate disease or those with relatively stable early perfusion require rapid fluid replacement. Additionally, challenges remain in implementing truly individualized, goal-directed strategies. These include high subjectivity and reliance on clinical experience, the absence of standardized quantitative indicators, and limited validation data for perfusion monitoring tools such as central venous pressure, lactate levels, and bedside ultrasound in patients with pancreatitis. To address these gaps, future research should involve larger-scale prospective studies stratified by patient phenotypes (e.g., obesity, HTG-AP) to better understand differences in fluid type responsiveness and optimal infusion rates. Furthermore, developing an early prediction model for volume responsiveness—incorporating biomarkers and imaging modalities—may help minimize fluid-related iatrogenic complications while ensuring adequate tissue perfusion. Ultimately, the goal is to establish a precision fluid resuscitation framework that enhances the safety and efficacy of fluid management in AP patients.

#### Nutritional support

Nutrition plays a critical role in the management of patients with AP. In the cases of MSAP and SAP, a robust systemic inflammatory response is often triggered, resulting in a catabolic state that substantially increases caloric and nutrient demands. Early nutritional support, particularly enteral nutrition, alleviates adverse effects by increasing caloric expenditure, increasing visceral blood flow to maintain intestinal mucosal integrity, and promoting intestinal motility.^377^ The benefits of enteral nutrition in AP have been consistently supported by evidence-based studies. Traditional practice often advocates fasting to allow pancreatic rest, on the basis of concerns that enteral feeding might stimulate pancreatic secretion and exacerbate inflammation. However, this view has been challenged by multiple studies.^378,379^ A Cochrane meta-analysis confirmed that, compared with parenteral nutrition, enteral nutrition significantly reduces mortality, the incidence of multiple organ failure, and the risk of systemic infections, with the mortality benefit particularly evident in critically ill patients.^380^

Furthermore, existing evidence consistently indicates that parenteral nutrition is not only more costly and associated with a higher risk of complications but also significantly less effective than enteral nutrition in terms of clinical outcomes.^10,125,381^ Therefore, in clinical practice, enteral nutrition should be the first-line approach for patients with AP, provided that gastrointestinal function is preserved. Parenteral nutrition should be initiated only within 72 h if there is an absolute contraindication to enteral feeding. When parenteral nutrition is needed, supplementation with 0.20 g/kg L-glutamine per day is recommended. Otherwise, immunonutrition does not appear to offer clinical benefit in the management of SAP.^382^ With respect to the timing of nutritional intervention in AP, current guidelines and clinical studies have reached a broad consensus. Most authoritative organizations recommend initiating enteral nutrition within 24–72 h after disease onset.^366,367,383^ Notably, early studies suggested that very early nasogastric feeding (within 24 h) did not demonstrate superiority over on-demand feeding starting after 72 h.^144^ However, a recent meta-analysis including seven RCTs confirmed that starting enteral nutrition within 24 h of admission reduces the risk of multiple organ failure compared with delayed initiation.^384^ Enteral nutrition modalities include oral feeding and nasoenteral feeding via nasogastric or nasojejunal tubes. Oral intake is preferred when feasible; however, for patients who are unable to eat by mouth within 72 h, timely initiation of nasoenteral nutrition is recommended. Although existing evidence suggests that nasojejunal feeding theoretically reduces pancreatic secretion, RCTs and meta-analyses have demonstrated that nasogastric and nasoduodenal tubes are clinically equivalent.^385,386^ For patients with severe or necrotizing pancreatitis requiring tube feeding, the AGA recommends selecting nasogastric or nasoenteric routes on the basis of individual clinical circumstances.^366^ Concerning nutritional formulas, there is currently insufficient evidence to support the superiority of elemental or semi-elemental diets over standard polymeric formulas (the potential role of TCM components will be discussed later). However, in patients exhibiting malabsorption symptoms such as steatorrhea, treatment should proceed cautiously. Exocrine pancreatic function should be assessed via fecal elastase-1, fecal fat analysis, or direct pancreatic function tests. In such cases, semi-elemental formulas containing predigested nutrients combined with pancreatic enzyme replacement therapy should be considered.^382^ These guidelines emphasize effective nutritional support while considering clinical practicality and individualized treatment.

However, several issues in clinical practice warrant further discussion and refinement. While early enteral nutrition has demonstrated clear benefits in MSAP to SAP, its role in MAP remains uncertain. Currently, there is insufficient evidence to determine the relative advantages or disadvantages of elemental, semi-elemental, or polymeric formulations. Moreover, the evidence base for nutritional support using TCM remains in the exploratory phase, and caution is advised regarding its empirical use. Importantly, in SAP, nutritional management should not focus solely on caloric supplementation but should also consider its anti-inflammatory, antioxidant, and immunomodulatory properties as part of a comprehensive therapeutic approach. Therefore, there is a need to establish a more precise and reproducible nutritional intervention model to fully realize the potential of nutritional support in promoting multi-organ protection and long-term recovery in AP.

#### Pain management

Abdominal pain is the most prominent clinical symptom of AP and may be correlated with disease severity.^387^ Pain relief is an important goal of clinical treatment, and adequate and effective analgesia not only relieves patient discomfort but also may help halt the progression of necrosis by improving pancreatic microcirculation.^388^ The stepwise analgesic approach recommended by the World Health Organization (WHO) remains the clinical gold standard for managing AP pain.^389^ This protocol advocates starting with non-opioid agents such as acetaminophen and escalating to weak or strong opioids on the basis of the analgesic response. This progressive strategy balances pain control with minimizing opioid dependence risk. Notably, regimens including acetaminophen have shown clinical benefit in critically ill patients, reducing mortality risk in those with APACHE III scores >83 and morphine doses >60 mg/day. Although this may prolong the duration of hospital stay, its long-term impact requires further investigation.^390^ In addition, selective cyclooxygenase-2 inhibitors, a subclass of nonsteroidal anti-inflammatory drugs, have demonstrated effective analgesic properties in single-center, open-label randomized controlled trials. These agents may help predict the likelihood of disease progression and have been associated with reduced local complication rates and disease severity.^391^ However, these findings require further validation in double-blind, multicenter trials. For critically ill patients, clinicians may consider adjusting the escalation or tapering of analgesic regimens on the basis of individual needs, especially in those experiencing severe pain or presenting contraindications to nonsteroidal anti-inflammatory drugs.^389^ Preliminary studies suggest that opioids and non-opioid analgesics are generally comparable in terms of overall safety,^392,393^ although opioids may offer unique advantages in reducing the need for rescue analgesia.^393^ For example, a recent RCT reported that buprenorphine outperforms diclofenac, significantly reducing fentanyl requirements, prolonging pain-free intervals, and improving VAS scores, with similar safety profiles, highlighting its potential as a first-line analgesic.^394^ However, recent evidence suggests that opioid use extending beyond six days after hospital admission may be associated with increased disease severity,^395^ highlighting the importance of carefully managing the duration of analgesic use. The combined application of systemic and topical analgesia, as well as the integration of patient-controlled analgesia with multimodal approaches, has attracted increasing attention in clinical settings. These strategies not only help achieve effective pain relief but also may reduce overall opioid consumption.^396^ For example, several RCTs have reported that compared with intravenous analgesia, epidural anesthesia not only effectively alleviates pain but also enhances pancreatic arterial perfusion and improves clinical outcomes.^397,398^ Nevertheless, given the limited research evidence, the risks and benefits of epidural anesthesia should be carefully evaluated in clinical practice. Additionally, evidence regarding acupuncture and other analgesic interventions will be discussed in the following sections. These specialized analgesic methods expand the options for managing pain in AP patients, yet their clinical application demands further high-quality research support. The aforementioned findings offer valuable insights for tailoring pain management strategies in AP. Generally, analgesic therapy should consider the pharmacological properties of the drug, the severity of the patient’s condition, and the potential risks associated with treatment. Future research should focus on refining multimodal analgesia protocols and evaluating their effects on long-term outcomes.

#### Antibiotic therapy

The use of prophylactic antibiotics in the early management of AP remains controversial. Although antibiotics may theoretically reduce the overall infection rate,^391^ multiple well-conducted clinical trials and meta-analyses have consistently shown that they do not effectively prevent specific complications, such as pancreatic infections, pneumonia, or urinary tract infections, nor do they reduce the need for invasive interventions or mortality.^399,400^ Current guidelines clearly state that prophylactic antibiotics should not be routinely administered for any form of AP unless there is definitive evidence of infection.^10,92,125,366^ For example, antibiotic therapy should be reserved for two well-defined scenarios: microbiologically confirmed infections or highly suspected infections—such as gas in necrotic areas, bacteremia, sepsis, or clinical deterioration.^401^ When treatment is indicated, broad-spectrum intravenous antibiotics capable of penetrating pancreatic necrosis—such as carbapenems or quinolones in combination with metronidazole—should be prioritized.^401^ If prophylactic antibiotics are deemed necessary, their use should be limited to 7–14 days; in cases of confirmed infection, therapy should be adjusted in accordance with the most recent critical care guidelines.^402^ Nonetheless, a substantial gap remains between clinical practice and guideline-based recommendations, with many patients still receiving unnecessary prophylactic antibiotics despite a lack of supporting evidence.^403,404^ This discrepancy underscores the persistent challenge of aligning clinical decision-making with evidence-based practices. For patients with suspected IPN, empiric antimicrobial therapy may be initiated, but culture and susceptibility testing should be performed promptly to allow for timely adjustment of the antimicrobial regimen.^92,401^

#### Etiological management

Surgical intervention remains the primary strategy to prevent the recurrence of biliary pancreatitis. For patients with mild disease, multiple studies have confirmed that cholecystectomy performed during initial hospitalization reduces the risk of biliary complications and significantly improves prognosis compared with delayed surgery (25–30 days after discharge).^405^ Delaying surgery beyond several weeks may increase the recurrence risk to as high as 30%.^406,407^ The AGA recommends completing surgery before discharge in mild cases.^92^ Patients with severe or necrotizing pancreatitis require individualized evaluation, and surgery is generally advised to be postponed until at least 8 weeks after onset to allow resolution of inflammation and to provide a clearer surgical field.^405,408^ For patients who are not surgical candidates, endoscopic biliary sphincterotomy may serve as an alternative; however, it only partially reduces the risk of pancreatitis recurrence and has limited efficacy in preventing acute cholecystitis or biliary colic.^408^ The use of ERCP remains controversial: the AGA recommends against routine emergency ERCP in patients without cholangitis,^366^ whereas the American College of Gastroenterology suggests prioritizing medical management over early ERCP intervention within 72 h.^92^

Alcohol-related AP is an independent risk factor for disease recurrence and progression to CP,^409^ with recurrence rates reaching up to 50% in affected patients, whereas abstainers have a significantly lower risk of recurrence.^410^ For this high-risk population, the AGA recommends a stepped intervention strategy: brief alcohol abstinence counseling during hospitalization, followed by intensive educational sessions every six months for two years after discharge. Systematic alcohol abstinence interventions effectively reduce recurrence; however, their clinical implementation remains suboptimal. Since both alcohol use and smoking are common risk factors for pancreatitis, smoking cessation interventions also hold important preventive potential.^366,411^

The treatment strategy for HTG-AP has evolved from traditional complete fasting to a more individualized, comprehensive approach. Specifically, stratification on the basis of serum triglyceride levels is key to clinical decision-making. For patients with severe HTG (triglyceride >11.3–22.6 mmol/L), fasting or insulin therapy can yield satisfactory outcomes.^412^ Plasmapheresis is recommended for patients with very severe HTG (triglycerides >22.6 mmol/L). Dual-filter plasma exchange, in particular, can reduce circulating triglyceride levels by 50–80% in a single session and has been shown to significantly alleviate systemic inflammation, making it the preferred approach in many centers.^413–416^ However, plasmapheresis carries a risk of allergic reactions, for which prophylactic antihistamines and glucocorticoids are often advised.^417,418^ Despite its rapid lipid-lowering effects, it has not been shown to reduce the incidence of organ failure and may increase the need for ICU admission.^419^ Insulin/heparin therapy has demonstrated comparable efficacy to plasmapheresis while posing fewer risks and reducing overall treatment costs.^420^ In exceptional cases where triglyceride levels exceed 133 mmol/L, plasmapheresis combined with continuous venovenous hemofiltration has been reported to markedly improve symptoms within 24 h. Although the precise mechanism remains unclear, this approach has gained wide clinical use.^421,422^ Long-term management of HTG is essential to prevent the recurrence of HTG-AP. Clinicians should closely monitor patients’ metabolic status following the acute phase and implement individualized management strategies. Lifestyle interventions should prioritize weight loss through dietary modifications—particularly reducing the intake of high-fat and high-sugar foods—and regular physical activity and the strict avoidance of alcohol due to its adverse impact on lipid metabolism. With respect to pharmacotherapy, fibrates (e.g., fenofibrate) are recommended as first-line agents for their potent triglyceride-lowering effects. Statins, niacin, and omega-3 fatty acids may serve as adjunctive options.^411^ Despite these advances, the management of HTG-AP remains challenging. Future research should aim to establish standardized treatment protocols based on triglyceride thresholds to reduce variability in clinical decision-making and better define the indications and timing for different blood purification strategies.^412,422^

### Late treatment of acute pancreatitis

The “step-up approach” serves as a guiding principle in the later-stage management of AP.^423^ This strategy primarily addresses local complications, with IPN being one of the most serious. Approximately 20–30% of patients with necrotizing pancreatitis develop secondary infection, which is associated with a mortality rate of approximately 30%.^424,425^ Historically, treatment for IPN has focused on open surgical debridement. However, in recent years, clinical practice has shifted toward more conservative management. Premature surgical intervention is now avoided in cases of sterile necrosis. Even when infection is present, a “delayed intervention” strategy—favoring endoscopic or minimally invasive approaches—is recommended. This delay allows necrosis to become better demarcated, helping to reduce procedural invasiveness and the risk of complications.^426,427^

#### Percutaneous catheter drainage

Percutaneous catheter drainage (PCD) involves the use of the Seldinger technique to puncture encapsulated IPN under ultrasound or CT guidance, followed by the placement of a drainage catheter to evacuate liquefied necrotic material. This approach helps relieve pressure within the abscess cavity and reduces the translocation of bacterial toxins into the bloodstream, thereby lowering the risk of SIRS and improving the patient’s overall clinical condition.^428,429^ As a “minimally invasive” intervention, PCD is effective in controlling the source of infection and delaying the need for definitive surgical procedures (e.g., necrosectomy), allowing the necrotic collection to mature into a WON. This delay significantly reduces intraoperative complications and decreases mortality.^429^ Furthermore, PCD can facilitate subsequent percutaneous interventions, such as percutaneous endoscopic necrosectomy or video-assisted retroperitoneal debridement. Initial drainage expands the necrotic cavity, making it possible to remove organized necrotic debris through the established tract.^430,431^ Importantly, PCD requires preprocedural planning of the puncture site and access path under imaging guidance to achieve effective multi-tract or “bridging” drainage. When feasible, the retroperitoneal approach is preferred to minimize the risk of infection spreading to the peritoneal cavity.^431^

#### Endoscopy

Endoscopic intervention mainly includes EUS-guided transgastrointestinal puncture and drainage and endoscopic necrotic tissue removal. For WON or pancreatic pseudocysts close to the stomach or duodenum, endoscopic drainage has a unique advantage: the establishment of an internal drainage channel through the gastrointestinal wall allows necrotic fluid to drain into the gastrointestinal tract without the need for superficial incisions, reducing the risk of external fistula and incisional infection.^432,433^ Current guidelines recommend that endoscopic interventions should be considered for patients who have failed conservative management and who have aseptic WON with compression of adjacent organs (e.g., the biliary tract or gastrointestinal tract) or who persist in pain 4 weeks after the onset of AP.^434^ For infectious necrosis, the purely endoscopic “transluminal” drainage-debridement strategy is comparable to surgical approaches and is even superior in terms of complication rates.^435^ For example, in the treatment of PFC drainage, double pigtail plastic stents and lumen-apposing metal stents (LAMSs) have shown high technical success rates and similar rates of adverse events, although the clinical success rate of the LAMS group was higher and the dependence on percutaneous drainage was lower. There was no significant difference between the two groups in terms of the recurrence rate or need for reintervention or surgery.^436,437^ Compared with 15-mm LAMS, 20-mm LAMS demonstrates similar clinical success and safety while requiring fewer endoscopic necrosectomy sessions to achieve WON resolution.^438^ With ongoing technical advancements, LAMSs equipped with electrocautery-enhanced delivery systems (EDS-LAMSs) have been increasingly adopted for EUS-guided PFC drainage in routine clinical practice. A large multicenter international study reported that EDS-LAMS is both safe and effective for various types of PFC.^439^ Therefore, endoscopic drainage or debridement is often the preferred minimally invasive approach in centers where it is available. This technique not only helps eliminate the source of infection but also maximizes the preservation of pancreatic tissue (avoiding over-resection), thereby improving patients’ quality of life. Endoscopic intervention can also complement percutaneous drainage; in certain cases, both endoscopic and percutaneous “dual-route” drainage can be employed to manage complex, multilocular necrotic collections, thereby increasing the overall success rate.^440,441^ However, the feasibility of endoscopic procedures depends heavily on advanced equipment and operator expertise, which may limit their availability in some hospitals. Anatomically, endoscopic access to necrotic cavities is considered safe only when the collection is in direct contact with the gastrointestinal wall and lacks internal septations. In contrast, abscesses located at a distance from the digestive tract (e.g., in the pelvis or adjacent to the left psoas muscle) are generally inaccessible via endoscopy and may require percutaneous or surgical approaches instead.

#### Surgical management

Necrotic pancreatic tissue can be removed through either open surgery (laparotomy) or minimally invasive approaches. Minimally invasive necrosectomy allows direct visualization of the necrotic area via laparoscopy, with tissue debridement performed via either hand-assisted or fully laparoscopic techniques. Compared with traditional open surgery, these minimally invasive methods are associated with reduced systemic inflammation and lower physiological stress, offering a more favorable recovery profile for selected patients. Various modalities—including laparoscopy, rigid nephroscopy, and flexible endoscopy—have been reported for this purpose. For instance, minimal access retroperitoneal pancreatic necrosectomy can help contain the spread of infection within the retroperitoneal space and is particularly suitable for retrogastric necrosis extending into the left paracolic gutter. This procedure may also be performed under video guidance, known as video-assisted retroperitoneal debridement, a technique first introduced by Horvath in 2001.^442^ However, video-assisted retroperitoneal debridement has certain limitations, including a restricted surgical field and limited visibility, often necessitating multiple sessions. Its applicability in cases of extensive necrosis—especially when extending to the right of the mesenteric vessels—remains controversial.^401^ Gastric debridement—via endoscopy, laparoscopy, or open surgery—is generally preferred for centrally located necrosis. However, when necrosis extends into the paracolic gutters, complete clearance may be difficult to achieve.^401^ Surgical transgastric debridement with internal drainage offers more efficient necrotic tissue clearance than endoscopic approaches do, likely because of the creation of a larger cystogastrostomy opening. In the MISER trial reported by Bang et al., 26 of 32 surgical patients (81.3%) underwent cystogastrostomy, while three patients were converted to endoscopic management because of intraoperative cardiovascular instability or technical failure. The rate of enteric or pancreaticocutaneous fistula was significantly greater in the surgical group (28.1%). Other reported complications included intraoperative bleeding (3 cases), incisional hernia (6.3%), and surgical site infection (6.3%).^443^ Laparoscopic transperitoneal debridement employs a traditional intraperitoneal approach. Its primary drawbacks include the risk of peritoneal contamination and the difficulty of performing reinterventions. Therefore, this method is generally considered suitable as a single-stage procedure for patients with WON.

In complex cases involving extensive necrosis and multiple non-communicating septated abscesses, open surgery may be required to perform comprehensive, multi-plane debridement along with irrigation and drainage—an outcome that is often difficult to achieve through a single minimally invasive approach. For certain critical emergency indications—such as progressive organ failure due to abdominal compartment syndrome, rupture and hemorrhage of a large pseudoaneurysm unresponsive to interventional treatment, or perforation of hollow viscera—timely surgical intervention often represents the final life-saving option. In these scenarios, surgery can rapidly relieve intra-abdominal hypertension or resect and repair bleeding lesions, playing an indispensable role in acute care. This surgical method involves the removal of devitalized peripancreatic and intrapancreatic tissue, along with drainage of fluid collections.^444^ In cases of extensive necrosis involving the retrocolic and/or mesenteric spaces where complete necrosectomy cannot be achieved in a single procedure, open packing or staged laparotomy may be employed as a damage control strategy.^445^ However, this approach has been associated with high complication rates in the literature, with a reported mortality rate of 24%, and often necessitates multiple operations.^446^ Compared with open packing or staged laparotomy, debridement with closed packing, another form of open necrosectomy, has been shown to reduce the need for reoperation. According to J. Ruben Rodriguez et al., this method is associated with a mortality rate of 11.4%, a reoperation rate of 12.6%, and a postoperative percutaneous drainage rate of 29.9%.^447^ However, surgery is a highly invasive procedure that has a great physiological impact on patients, and surgery at the peak of systemic inflammation in pancreatitis often exacerbates inflammation and organ dysfunction. In addition, traditional open necrosis resection often requires multiple secondary operations or continuous irrigation of the open abdominal cavity, which affects the long-term quality of life of patients.

## Adjuvant therapy with traditional Chinese medicine

In recent years, TCM has played an important role in the adjuvant treatment of AP, significantly improving clinical efficacy, reducing the burden of disease, and providing clinicians with more options (Table 4).^448^

**Table 4:** Traditional Chinese medicine approaches for acute pancreatitis

TCM treatment modality	Intervention stage	Advantages	Limitations
TCM and its active monomers	Applicable to early and severe stages of AP	Exert a multi-target role of anti-inflammation, immune regulation and intestinal mucosal protection	Lack large-scale randomized controlled trials
TCM formulas	Applicable throughout the disease course; treatment can be adjusted based on the course of disease	Synergistic effects of multiple components; suitable for AP with complex pathophysiology	Complex composition and unknown mechanism; low quality clinical research
Enema treatment	Applicable to early AP and intestinal dysfunction	Provide a local delivery route for fasting or intestinal dysfunction; facilitate targeted removal of intestinal stasis and inflammatory mediators	Not suitable for patients with severe shock or frequent diarrhea; prolonged retention may cause discomfort and poor compliance
Acupuncture treatment	Applicable throughout the disease course	Alleviate inflammation and acute symptoms in early AP; improve gastrointestinal function and reduce enterogenic infection risk during recovery	Efficacy is operator-dependent with risks such as bleeding and infection; most clinical studies are single-center, small-sample studies with limited validation
Point application	Applicable to the stable or recovery phase	Easy to administer and non-invasive	Limited efficacy when used alone; may cause skin irritation or allergy

*AP* acute pancreatitis, *TCM* traditional Chinese medicine

### Traditional Chinese medicine and its active monomers

According to TCM, AP is categorized as a condition resulting from “intermingled phlegm and blood stasis”. Rhubarb, known for its laxative, heat-clearing, and blood-invigorating properties, has long been regarded as a key therapeutic agent in the treatment of AP.^449^ Modern pharmacological studies have further demonstrated that rhubarb can inhibit the MAPK signaling pathway, thereby alleviating AP-related pathological damage.^450^ Emodin, the primary bioactive compound extracted from rhubarb, also has significant protective effects against AP. For example, it helps preserve the integrity of the peripancreatic barrier and supports pancreatic function.^451^ In vitro experiments have shown that emodin attenuates sodium taurocholate-induced pancreatic injury by lowering the levels of inflammatory cytokines, such as IL-1β and TNF-α, and suppressing the release of ROS.^452^ In addition to exerting a protective effect on the pancreas, emodin exerts synergistic protective effects on extrapancreatic organs, including the lungs and intestines. Specifically, emodin has been reported to reduce neutrophil infiltration in lung tissue,^453^ and inhibit extracellular collagen matrix degradation,^454^ thereby mitigating lung injury. Our previous study also indicated that emodin reduces alveolar macrophage pyroptosis and alleviates AP-induced lung tissue damage.^455^ Moreover, plasma-derived exosomes (Exos) from emodin-treated rats with SAP were found to suppress the proinflammatory polarization of alveolar macrophages by modulating the NF-κB signaling pathway, thereby attenuating lung inflammation.^331^ In terms of intestinal involvement, early research suggested that rhubarb may protect the intestinal mucosal barrier by regulating the gut microbiota and modulating TLR signaling.^456^ Similarly, emodin has been shown to reduce inflammatory mediator levels,^457^ inhibit the apoptosis of intestinal epithelial cells.^458^ Together, these findings not only deepen our understanding of the multifaceted actions of emodin in AP but also highlight potential therapeutic targets and strategies for future interventions. In addition, various TCM compounds have demonstrated encouraging therapeutic potential in studies on AP. For example, red peony root has been shown to alleviate clinical symptoms in patients with MSAP and SAP by modulating the IL-6 and TNF-α inflammatory pathways.^459^ This improvement is reflected in shorter durations of fever and abdominal pain, earlier recovery from spontaneous intestinal peristalsis, lower modified Balthazar CT scores, and reduced levels of serum inflammatory markers. Other TCM-derived monomers, including naringin,^460^ ganoderic acid A,^461^ and *Tephrosia purpurea*,^462^ have also shown organ-protective effects in SAP models.

As research continues to evolve, combining individual monomers appears to achieve synergistic benefits across multiple dimensions, including inflammation control, immune modulation, and organ protection. This “1 + 1 > 2” effect provides a broader therapeutic scope for AP treatment. For example, the combination of emodin and baicalin has been reported not only to inhibit leukocyte chemotaxis^463^ but also to confer multi-target protection by regulating calcium signaling and cytokine networks.^464^ These findings reflect a growing trend in TCM research toward multi-component, multi-target therapeutic strategies. In addition, owing to their high purity and well-defined mechanisms of action, TCM monomers are often employed as adjunctive therapies during the early or severe stages of AP. However, robust clinical evidence for many of these agents remains limited, with most data derived from small-scale studies or empirical use. The therapeutic effects of individual components tend to be localized and may not fully address the complex pathophysiology of AP; thus, combination or formula-based interventions are often needed for optimal benefit.

### Traditional Chinese medicine formulas

In addition to monomeric components, TCM formulas also have distinct therapeutic potential in the treatment of AP. Da Cheng Qi Decoction (DCQD), a representative formula of the “Tongfu Xiexia” method, exerts multi-target and multi-organ protective effects, particularly in SAP. Its mechanisms include reducing acinar cell necrosis, promoting apoptosis, regulating barrier function, and suppressing inflammatory signaling pathways.^465^ Clinical studies have further shown that DCQD can significantly lower mortality in SAP patients and reduce the incidence of MODS.^466^ Additionally, DCQD attenuates kidney inflammation by modulating inflammatory cytokines,^467^ improves intestinal function via the regulation of barrier proteins,^468^ and reduces serum levels of HMGB1, thereby alleviating AP-ALI,^469^ reflecting its systemic protective profile. In summary, DCQD shows promising clinical potential in AP/SAP treatment via a multi-component, multi-target mechanism, although further high-quality randomized controlled trials are needed for validation. Chaiqin Chengqi Decoction (CQCQD), a derivative of DCQD, has also exhibited favorable therapeutic potential in AP. Clinical studies have reported that CQCQD reduces the serum levels of multiple proinflammatory cytokines in AP patients.^470,471^ In cases of severe acute biliary pancreatitis, CQCQD significantly decreases morbidity and mortality and shortens hospital stays,^472^ supporting its clinical applicability. Mechanistically, CQCQD helps maintain calcium homeostasis in pancreatic acinar cells^473,474^ and activates the cholinergic anti-inflammatory pathway,^475^ thus mitigating pancreatic injury. Moreover, CQCQD offers unique advantages in HTG-AP by modulating key enzyme activity in the glycerophospholipid metabolic pathway.^476^ It also has the potential to reduce distal organ injury, such as mitigating pulmonary vascular endothelial hyperpermeability induced by endotoxemia.^477^ Collectively, the current findings suggest that CQCQD has extensive anti-inflammatory and organ-protective effects on AP.

Clinical practice and multiple studies have demonstrated that various herbal combinations have been applied in the treatment of AP. These formulas act synergistically, targeting multiple pathological pathways and offering distinct therapeutic benefits at different disease stages. For example, Qingyi decoction has been shown to reverse SAP-induced dysbiosis of the intestinal microbiota,^478,479^ thereby protecting against intestinal damage in SAP. Similarly, Shengjiang decoction reduces the serum levels of amylase and TNF-α, upregulates IL-10, and improves multi-organ pathological injury in AP rats.^480^ In addition, Chaihuang Qingfu pills have been shown to lower the incidence of AP-ALI by inhibiting MMP9/NLRP3 pathway-mediated pyroptosis.^481^ Our group also reported that Zengye decoction can ameliorate SAP-related acute kidney injury by modulating the “microbiota–metabolite axis”.^482^

Therefore, TCM formulas may be particularly well suited for diseases with complex pathogenesis, such as AP. Both clinical and experimental studies have supported their ability to reduce inflammation, preserve organ function, and facilitate recovery. In clinical practice, compound prescriptions can be flexibly adjusted on the basis of the stage of disease progression. During the acute phase, treatment emphasizes heat clearance and bowel purgation; in the subacute phase, the focus shifts to removing blood stasis and detoxification; and in the recovery phase, the aim is to strengthen the spleen and replenish qi, thereby providing stage-specific therapeutic optimization. However, timing remains critical in combination therapy. Delayed intervention may miss the optimal window for controlling inflammatory peaks, and treatment strategies should be adjusted according to individual tolerance. Notably, TCM compounds consist of multiple herbal components, and their mechanisms of action are relatively complex. Challenges such as ingredient variability, quality control, and limited international acceptance persist. While numerous clinical studies in China have reported positive effects of compound formulas, many are constrained by small sample sizes, lack of double-blind designs, and limited evidence levels. Future research should aim to validate the clinical value of TCM compounds through large-scale, rigorously designed, and high-quality evidence-based studies. These efforts will help advance their application in AP treatment and facilitate the translation of basic research into clinical practice.

### External therapies involving traditional Chinese medicine

TCM external therapies offer distinctive supportive benefits in managing AP, including acupuncture, acupoint application, and enema therapy. Acupuncture can modulate autonomic nervous activity and attenuate inflammatory responses by stimulating specific acupoints,^483^ making it a potentially valuable adjunct for SAP. It may help lower the risk of MODS and improve clinical outcomes, although its effectiveness is closely tied to the practitioner’s expertise. Inappropriate acupoint selection or suboptimal technique may limit benefits, and it can be challenging to perform in severely debilitated or uncooperative patients. While the overall risk of adverse events such as bleeding or infection is relatively low, careful attention to indications remains essential. Enema therapy (e.g., Hu‒Pi‒Cheng‒Qi decoction) facilitates the clearance of intestinal stasis via rectal administration and can reduce circulating inflammatory cytokine levels.^484^ When applied early, it may help mitigate excessive inflammatory responses; however, standardized protocols are needed to minimize the risk of mucosal injury. Acupoint application (e.g., Liu He Dan, mirabilite) exerts anti-inflammatory effects^485^ and alleviates tissue edema^486^ through transdermal absorption. This approach is typically recommended during the stable or recovery phase of AP and may provide supportive benefit, though it is not a substitute for core treatment strategies. Skin irritation from herbal patches can occur, and maintaining skin integrity during use is important.

Overall, the above TCM-based therapies demonstrate distinct advantages at different stages of AP and may achieve improved efficacy when combined with conventional supportive care. Importantly, in severe stages, such as SAP, TCM therapies should be used in conjunction with modern intensive care practices and cannot replace essential interventions such as fluid resuscitation, nutritional support, and life-sustaining measures. Maximizing the therapeutic benefits of TCM in AP requires appropriate timing and adherence to the principles of syndrome differentiation.

## Treatment strategies for pancreatitis in special populations

The treatment of AP during pregnancy should be individualized on the basis of the underlying etiology and the patient’s specific clinical characteristics. In recent years, the incidence of acute biliary pancreatitis during pregnancy has increased, likely related to increased maternal age, increased obesity rates, and increased prevalence of comorbidities. While overall maternal mortality remains low, the increasing occurrence of SAP—especially in individuals with multiple comorbid conditions and among certain ethnic groups—warrants increased clinical attention. Greater adherence to evidence-based interventions, including ERCP and cholecystectomy, has been associated with a reduced hospital length of stay in routine care. Future studies should focus on addressing disparities in healthcare access and optimizing management approaches to alleviate the clinical burden of SAP and support more equitable outcomes for both mothers and newborns.^487^ In cases of HTG-induced AP during pregnancy, standard supportive care remains essential; however, a rapid reduction in triglyceride levels—preferably below 5.65 mmol/L—is a key therapeutic goal.^488^ Octreotide, a somatostatin analog used to suppress pancreatic enzyme secretion, is capable of crossing the placental barrier. However, safety data during pregnancy are limited primarily to isolated case reports and small series, and robust evidence is lacking.^489,490^ Similarly, the safety profile of ulinastatin in pregnancy is supported by case reports, such as those of a 36-year-old woman treated at 11 weeks of gestation who subsequently delivered a healthy infant at term.^491^ Nevertheless, additional data are needed to enable a more comprehensive risk assessment. The use of lipid-lowering agents during pregnancy necessitates a cautious evaluation of potential risks and benefits. Clinical experience with the use of fenofibrate in human pregnancy remains limited. Although available case reports—primarily involving exposures during late gestation—have not indicated an association with congenital anomalies, evidence regarding first-trimester exposure is particularly sparse.^492,493^ Ezetimibe has been described in only two reported pregnancy cases—one during early pregnancy and one during mid-gestation—both without adverse outcomes; however, the sample size is too small to draw definitive conclusions.^494^ Notably, in 2021, the United States FDA lifted the contraindication for statin use during pregnancy for high-risk patients, such as those with familial hypercholesterolemia, although routine use remains discouraged.^495^ In addition, insulin enhances lipoprotein lipase activity, thereby accelerating chylomicron clearance, whereas heparin promotes the release of lipoprotein lipase from endothelial cells—both of which contribute to triglyceride reduction.^496^ However, caution is warranted: insulin may induce hypoglycemia in non-diabetic patients, and prolonged use of heparin can lead to the depletion of circulating lipoprotein lipase stores. Overall, the management of HTG-AP during pregnancy remains inadequately standardized, with most treatment decisions guided by observational data. When the clinical condition is critical and the potential benefits outweigh the risks, cautious use of the above agents may be considered. Multidisciplinary collaboration and close maternal–fetal monitoring remain essential components of care.

Compared with adults, the direct application of adult treatment protocols to pediatric patients raises several concerns. For example, in fluid resuscitation, pediatric fluid requirements must be tailored to body weight, and the recommended infusion rates differ physiologically from those for adults.^497^ Current studies suggest administering 1.5 to 2 times the maintenance fluid volume within the first 24 h of admission, with close monitoring of urine output. This approach appears to be safe and has been associated with favorable outcomes.^497^ However, multicenter studies are still needed to determine the optimal fluid composition, therapeutic endpoints, and monitoring strategies. In early research on nutritional support for pediatric AP patients, some studies reported that early oral feeding significantly reduces the length of hospital stay.^498^ Similarly, enteral nutrition initiated within 48 h has been linked to shorter hospitalization durations.^499^ A meta-analysis of these two studies confirmed the superiority of early nutrition over fasting.^500^ Nevertheless, other studies reported no significant difference in the length of stay between early- and delayed-feeding groups.^501,502^ A recent study reported no differences in hospital stay, inflammatory marker levels, or pancreatic enzyme levels between extremely early feeding (within 24 h) and early feeding (after 24 h) in children with MAP.^503^ Further research is warranted to evaluate this strategy in more severe cases.^503^ Additionally, some studies have identified nutritional deficiencies, including reduced ferritin, low levels of fat-soluble vitamins, and hypoalbuminemia, in pediatric AP patients. In severe cases, decreased BMI Z scores, vitamin E deficiency, and an increased risk of abnormal glucose metabolism at 12 months have been observed.^504^ These findings suggest that long-term monitoring of nutritional status in pediatric AP patients is essential. Pain management practices for pediatric AP vary across regions. The North American guidelines recommend intravenous morphine when acetaminophen or non-steroidal anti-inflammatory drugs are insufficient.^505,506^ A Canadian survey indicated that 94% of pediatric gastroenterologists listed opioids as their first-line choice.^507^ In contrast, European guidelines reserve opioids for severe pain and recommend acetaminophen and ibuprofen as first-line agents.^508^ There is an urgent need for further research in underrepresented regions such as Asia and Africa to examine trends in analgesic selection and their associations with socioeconomic and clinical characteristics.^506^

## Emerging therapies and recent advances

### Nanotechnology

Nanotechnology, through the precise manipulation of materials at the scale of 1–100 nm, offers promising strategies for the treatment of AP.^509^ Compared with conventional dosage forms, nanocarriers and nanodrugs—due to their small particle size, high specific surface area, and excellent solubility—can penetrate the expanded vascular interstitial space at inflamed sites, overcome the blood‒pancreas barrier and cellular membrane barriers, and achieve targeted delivery to pancreatic tissue. These features improve drug safety, enhance tissue permeability, and optimize therapeutic outcomes.^510–512^ These advantages have made nanomaterials an emerging focus in AP research, offering new avenues for modulating inflammation and enhancing drug delivery efficiency. Natural compounds hold potential in AP therapy, yet their physicochemical limitations hinder their clinical application. Kaempferol, for example, exerts anti-inflammatory effects by modulating the LPS–TLR4–NF-κB and IRE1–JNK–CHOP pathways^513,514^ but is poorly soluble and stable.^515^ To address this, thioketone-modified liposomal nanocarriers (DTM@KA NPs) have been employed to significantly increase kaempferol bioavailability and alleviate pancreatic inflammation by promoting STAT6-mediated mitochondrial protein transport and Pink1/Parkin-regulated autophagy.^516^ Biomolecular carriers also present unique advantages in AP treatment. Compared with conventional carriers, their enzyme-responsive properties allow for more specific drug release.^517^ For example, silk protein nanoparticles release bilirubin in response to trypsin, attenuating oxidative stress and inflammation by inhibiting ROS and activating the Nef2/HO-1 pathway.^518^ In another study, neutrophil membrane-coated HMPB nanoparticles effectively targeted inflamed tissue and restored pancreatic function by regulating ER stress and autophagy via the Beclin-1/LC3 axis.^519^ More recently, a macrophage membrane-coated nanodelivery system was developed to increase the therapeutic efficacy of emodin against AP. Compared with free emodin, the macrophage membrane-coated nanodelivery system significantly reduced serum α-amylase and lipase levels,^520^ underscoring the precision of biological carrier-based strategies. In addition to the use of nanocarriers to encapsulate therapeutic agents, another promising approach involves the self-assembly of drugs into nanoparticles via external stimuli. Nano-selenium, for example, offers anti-inflammatory and antioxidant benefits owing to its simple synthesis and good biocompatibility.^521,522^ Yttrium oxide nanoparticles have been found to improve mitochondrial and ER function while downregulating inflammatory markers through Nrf2/NF-κB signaling.^523^ Tetrahedral framework nucleic acids exert therapeutic effects by reducing the levels of enzymes associated with AP and inhibiting pathological cell death.^524^ Furthermore, copper-based metal–organic framework nanozymes mimic superoxide dismutase and catalase activities to scavenge ROS and regulate mitochondrial autophagy, thus offering a novel therapeutic avenue for AP.^525^ Collectively, nanotechnology-based interventions have shown great potential in promoting AP therapy and deserve further research and development.

Although nanotechnology has shown promising progress in the context of AP, its clinical translation still faces significant challenges. First, there is a lack of systematic evaluation regarding the biosafety of certain nanomaterials, particularly the in vivo metabolism, accumulation, and immunogenicity of inorganic nanoparticles. Second, cell membrane-coating technologies have been explored to increase targeting, delivery efficiency, and target specificity. Off-target effects remain unstandardized and require further optimization, particularly through in vivo tracking and tissue distribution studies. In addition, the combination of natural compounds with nanosystems faces limitations such as low drug-loading capacity and unstable release profiles, highlighting the urgent need for the development of intelligent nanocarriers to improve delivery precision. Importantly, different animal models may also exhibit phenotypic variability in response to nanomedicines. Therefore, validation across multiple models, especially large animal models, is recommended to better assess efficacy and safety. Furthermore, standardization of formulation processes and translational research on industrial scalability are essential steps toward achieving early clinical application of nanomaterials.

### Extracellular vesicles

Extracellular vesicles (EVs) are nanomaterials that are lipid bilayers released by cells and present in a variety of biological fluids.^526,527^ They can directly interact with recipient cell membranes to deliver proteins, nucleic acids, lipids, and other cargo from their cells of origin, thereby participating in the regulation of biological processes and mediating intercellular communication.^528^ EVs have been implicated in a wide range of diseases. On the basis of their subcellular origin and size, EVs are commonly classified into three major categories: Exos, microvesicles, and apoptotic bodies.^529^ Among these EVs, Exos are the smallest, typically ranging from 30 to 120 nanometers in diameter.^530^ In recent years, an increasing number of studies have highlighted the critical role of Exos in AP, particularly in its pathogenesis, diagnosis, and potential therapeutic applications.

One of the earliest studies reported the presence of two distinct sources of Exos in a rat model of AP: one secreted by the pancreas and present in pancreatic ascitic fluid and the other released by the liver and found in plasma.^531^ These two Exo populations differ in both protein and miRNA contents. Compared with pancreatic ascitic fluid-derived Exos, plasma-derived Exos are enriched in inflammatory miRNAs such as miR-155 and miR-21. In contrast, pancreatic ascitic fluid Exos contain 10- to 30-fold higher levels of histone and ribosomal proteins than their plasma counterparts do,^531^ suggesting that Exos exert different physiological effects depending on their tissue of origin and site of action. For example, Yang et al.^532^ demonstrated that alveolar lavage-derived Exos from SAP rats treated with emodin exhibited a more pronounced therapeutic effect than did plasma-derived Exos in the same model. Additionally, EVs have been shown to contribute to AP progression through the polarization of proinflammatory macrophages. For example, EVs secreted by cerulein-stimulated acinar cells delivered miR-183-5p, which downregulated forkhead box protein O1, promoted M1 macrophage polarization, and increased macrophage infiltration into the pancreas, ultimately leading to acinar cell injury and trypsinogen activation.^533^ Additionally, in a rat model of obesity-related SAP, serum-derived Exos were found to stimulate the secretion of proinflammatory cytokines, including IL-1, IL-6, and TNF-α, and exacerbate pancreatitis severity by promoting M1 polarization of adipose tissue macrophages.^534^ Therefore, suppressing the proinflammatory polarization of pancreatic macrophages is considered essential for mitigating the onset and progression of pancreatitis, as discussed in the previous section. Moreover, our study revealed that serum-derived Exos from SAP model mice alleviate or prevent SAP-associated lung injury by targeting pulmonary microvascular endothelial barriers via the upregulation of the integrins ITGAM and ITGB2.^535^ Treatment with the HYD-1 polypeptide or engineered Exos that block the effects of ITGAM and ITGB2 effectively improved or prevented SAP-related lung injury.^535^

Since the pancreas can release EVs carrying pathological signals into the peripheral blood at early stages of the disease, serum EVs hold promise as early diagnostic biomarkers for SAP. For example, Lou et al.^536^ employed quantitative metabolomics to analyze plasma EV metabolite profiles in healthy controls, SAP patients, and those with MAP, identifying four metabolites—eicosatrienoic acid, thiamine triphosphate, 2-acetylfuran, and cis-citral—as potential SAP biomarkers. Moreover, alterations in serum EV-associated microRNAs may serve as predictors for SAP. For example, miR-483-5p and miR-503-5p have been proposed as candidate markers for SAP-associated ALI,^537^ whereas miR-21-5p is reportedly elevated in the serum EVs of patients with type 1 autoimmune pancreatitis and can help distinguish CP patients from healthy individuals.^538^ In addition, MSC-derived EVs also show potential for AP therapy since EVs derived from MSCs can induce angiogenesis, inhibit acinar cell necrosis, and promote pancreatic tissue regeneration. For example, EVs secreted by hair follicle-derived MSCs can improve the viability of caerulein-stimulated acinar cells and reduce the expression levels of pancreatic inflammation markers and key pyroptosis proteins in SAP mice, thereby improving AP inflammation.^539^ Furthermore, EVs are being explored as carriers for targeted drug delivery. For example, engineering EVs to load p88 has shown potential for selectively targeting pancreatic β cells,^527^ which may enhance therapeutic efficacy and represents a promising approach for the treatment of pancreatic diseases.

Therefore, injured acinar cells may contribute to the activation of macrophages or compromise the integrity of pulmonary endothelial barriers by releasing EVs that carry pathogenic signals, thereby promoting inflammatory responses and mediating damage to distant organs. Advances in nanomedicine have demonstrated that EVs can be engineered for the targeted delivery of therapeutic agents. However, their clinical translation faces several significant challenges. First, there is a lack of standardized protocols for EV purification and preparation. Second, issues related to the route of administration and biodistribution remain unresolved, highlighting the need for precise targeting strategies or localized delivery to increase therapeutic efficacy. Moreover, most current studies are based on small rodent models, with limited validation in large animal models or organoid systems. Additionally, systematic toxicological evaluations of EVs are lacking, and their bioactive molecules and specific targets remain to be fully elucidated. Moving forward, it will be essential to advance large-scale animal studies, clarify the underlying mechanisms, and optimize dosing regimens. Moreover, initiating early-phase clinical trials and establishing robust quality control systems will be critical for facilitating true clinical translation.

## Conclusion

In recent years, the incidence and mortality rates of AP have continued to rise, making it an increasingly urgent global public health concern. This growing burden underscores the need for more effective early interventions and comprehensive management strategies to reduce the progression to SAP and improve patient outcomes. In this context, the integration of AI has introduced new opportunities for risk stratification and prognostic evaluation in AP, with the potential to increase the accuracy and timeliness of clinical assessments and to support more informed therapeutic decision-making. Moreover, treatment strategies for AP have undergone significant evolution, shifting from traditional open surgical approaches toward more refined, stage specific, and personalized intervention models. TCM, as an important adjunctive therapy, has shown encouraging potential through its active monomers, multi-herbal formulations, and external treatment applications, highlighting the value of combining TCM with Western medicine in a complementary manner. Additionally, emerging therapeutic modalities—such as nanotechnology and EV–based therapies—have opened new avenues for precision treatment of AP. Looking ahead, continued investigation into the multilevel regulatory mechanisms underlying this condition, along with the development of more targeted and individualized treatment strategies, remains essential for improving clinical outcomes and facilitating the translation of personalized therapies into clinical practice.
